# Association of Plasma Metabolic Biomarker Sphingosine-1-Phosphate With Cerebral Collateral Circulation in Acute Ischemic Stroke

**DOI:** 10.3389/fphys.2021.720672

**Published:** 2021-08-19

**Authors:** Fang Yu, Xianjing Feng, Xi Li, Zeyu Liu, Di Liao, Yunfang Luo, Minping Wei, Qin Huang, Lin Zhang, Jian Xia

**Affiliations:** ^1^Department of Neurology, Xiangya Hospital, Central South University, Changsha, China; ^2^Clinical Research Center for Cerebrovascular Disease of Hunan Province, Central South University, Changsha, China; ^3^National Clinical Research Center for Geriatric Disorders, Xiangya Hospital, Central South University, Changsha, China

**Keywords:** acute ischemic stroke, metabolomics, sphinganine-1-phosphate, sphingosine-1-phosphate, collateral circulation

## Abstract

**Background:** The contribution of metabolic profile to the cerebral collateral circulation in acute ischemic stroke (AIS) has not been fully outlined. In this study, we conducted a metabolomic study to assess the relationship between the metabolic biomarkers and the collateral status of AIS.

**Methods:** A two-stage study was conducted from September 2019 to June 2021 in our hospital. There were 96 subjects including 66 patients with AIS and 30 healthy controls in the discovery stage and 80 subjects including 53 patients with AIS and 27 healthy controls in the validation stage. Collateral circulation was assessed by the Tan score based on computed tomographic angiography (CTA). Liquid chromatography-tandem mass spectrometry was used to identify differential metabolic markers. Then, an ELISA was employed to detect the plasma levels of sphingosine-1-phosphate (S1P).

**Results:**There were 114 differential metabolites between patients with AIS and control groups and 37 differential metabolites between good collateral circulation (GCC) and poor collateral circulation (PCC) groups. The pathway enrichment analysis revealed that arginine biosynthesis was the only statistically significant pathway between AIS and control groups and sphingolipid metabolism was the only statistically significant pathway between GCC and PCC groups. The differential metabolites sphinganine-1-phosphate (SA1P) and S1P belong to the sphingolipid metabolism. In the discovery stage, when the GCC group was compared with the PCC group, the receiver operating characteristic (ROC) analysis showed that plasma SA1P relative levels demonstrated an area under the curve (AUC) of 0.719 (95% CI: 0.582–0.834), and S1P levels demonstrated an AUC of 0.701 (95% CI: 0.567–0.819). In addition, both plasma SA1P and S1P relative levels showed significant negative correlations with the 90-day modified Rankin Scale (mRS) score. In the validation sample, higher plasma S1P levels were independent predictors of GCC (*p* = 0.014), and plasma S1P levels demonstrated an AUC of 0.738 (95% CI: 0.599–0.849) to differentiate patients with GCC from patients with PCC. In addition, plasma S1P levels also showed significant negative correlations with the 90-day mRS score.

**Conclusion:** We first illustrated the association between plasma metabolic profiles and cerebral collateral circulation in patients with AIS. Plasma S1P levels might be a potential diagnostic biomarker for predicting collateral circulation status in patients with AIS.

## Introduction

The cerebral collateral circulation status plays a vital role in protecting the brain against ischemia and reperfusion injury. Accumulating studies have shown that the well-developed collateral status independently predicted the favorable clinical outcomes in patients with acute ischemic stroke (AIS) in response to intravenous thrombolysis (IVT) or endovascular treatment (Bang et al., 2011; Menon et al., 2015).

Cerebral collateral circulation can be divided into primary (circle of Willis) and secondary collateral pathways (the ophthalmic artery and leptomeningeal vessels) (Liebeskind, 2003). Leptomeningeal collateral circulation is preexisting anastomoses that are crucial to the functional outcome of AIS, especially after mechanical thrombectomy (Lima et al., 2010). Clinically, available tools for the evaluation of collateral circulation are mostly based on imaging techniques like digital subtraction angiography, computed tomography perfusion, computed tomographic angiography (CTA), and some magnetic resonance-based methods. Although these imaging techniques play essential roles in differentiating collateral status, sometimes they are time-consuming, not cost-effective, and limited by some contraindications.

Collateral circulation varies greatly in patients, the reasons of which remain largely unknown. Several studies have shown that vascular risk factors like hypertension, metabolic syndrome, smoking, and aging were related to poor collaterals (Lima et al., 2010; Menon et al., 2013). However, the underlying molecular pathogenesis regulating the collateral circulation of the brain remains unclear. Biomarkers are crucial in the prediction and diagnosis of stroke and could also help improve our understanding of the etiology and pathophysiology of stroke, whereas only a few studies have explored the relationship between circulating biomarkers and collateral circulation status. Laura Mechtouff et al. found that high matrix metalloproteinase-9 (MMP-9) and low monocyte chemoattractant protein-1 (MCP-1) levels were associated with poor pretreatment collateral circulation in patients with stroke with large vessel occlusion (LVO) (Mechtouff et al., 2020). Another study demonstrated that higher plasma apelin-17 levels were positively associated with good collateral circulation (GCC) in patients with ischemic stroke (Jiang et al., 2019). Furthermore, plasma proteomic profiling of patients with AIS with LVO identified three collateral-related proteins, namely, IGF2, LYVE1, and THBS1 (Qin et al., 2019). However, none of these biomarkers entered reached clinical application.

Using an untargeted metabolomics based on liquid chromatography-tandem mass spectrometry (LC-MS) might help decipher potential metabolites involved in the stroke onset and progression and uncover novel pathophysiological processes of ischemic stroke. Since metabolomics can uncover small metabolites which could penetrate the blood–brain barrier, it might be an emerging powerful tool for exploring novel biomarkers of ischemic stroke. Currently, several circulating metabolites and pathways have been found associated with stroke risk predictions and diagnosis. However, less is known about whether metabolomics also plays a role in the cerebral collateral circulation status. In this study, we conducted an untargeted metabolomics study using LC-MS (AB SCIEX TripleTOF 5600) to identify differential metabolites in patients with AIS with GCC and poor collateral circulation (PCC).

## Materials and Methods

### Study Participants

**Figure 1:**
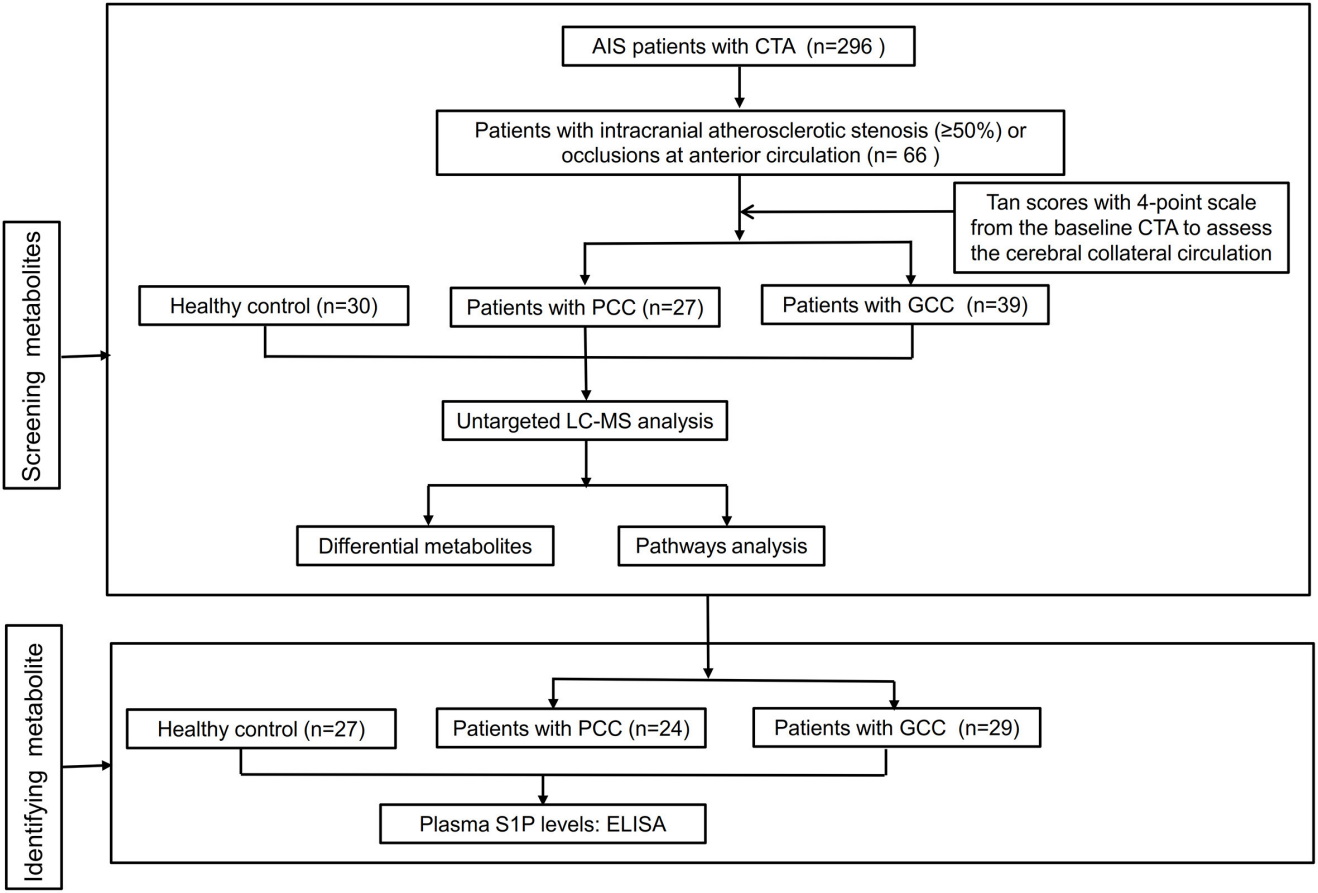
Flowchart of the study design. AIS, acute ischemic stroke; CTA, computed tomographic angiography; GCC, good collateral circulation; PCC, poor collateral circulation; LC-MS, liquid chromatography-tandem mass spectrometry; S1P, sphingosine-1-phosphate; ELISA, enzyme-linked immunosorbent assay.

A two-stage study was conducted from September 2019 to June 2021 in Xiangya Hospital, Central South University, and the flowchart of the study design is shown in Figure 1. A total of 119 patients with anterior circulation AIS (66 in the discovery stage and 53 in the verification stage) with intracranial atherosclerotic stenosis (≥50%) or occlusions within 7 days after symptom onset were prospectively enrolled in this study. All patients underwent brain MRI and diffusion-weighted imaging (DWI) scans. Patients with histories of stroke, autoimmune disease, malignant tumors, severe infection, severe hepatic, and renal dysfunction were excluded. During the same period, we recruited 30 and 27 healthy control subjects from the physical examination center of Xiangya Hospital in the discovery and verification stages, respectively. This study was approved by the Ethics Committee of Xiangya Hospital of Central South University, and all patients provided written informed consent. EDTA-containing blood samples of all patients were collected after fasting overnight and then centrifuged at 3,000 rpm for 15 min at 4°C. These plasma samples were stored immediately at −80°C until analyses.

### Image Acquisition and Imaging Analysis

All selected patients completed CTA examinations. We applied a previously reported Tan scoring system with a scale of 0–3 from the baseline CTA to assess the cerebral collateral circulation as follows: 0, absent collateral of the occluded vascular territory; 1, poor collateral filling (>0% but ≤ 50% of the occluded vascular territory); 2, moderate collateral filling (>50% but <100% of the occluded vascular territory); and 3, good collateral filling (100% of the occluded vascular territory) (Wiegers et al., 2020). Based on the collateral circulation scores, patients with AIS were categorized into the following two groups: GCC group with the Tan scores of 2–3 and PCC group with the Tan scores of 0–1. Two neurologists (FY and XJF) assessed the collateral status of patients with AIS together.

### Clinical Examination

We collected the demographic and clinical data of all participants from the medical record. After overnight fasting, blood count, total cholesterol (TC), triglycerides (TG), high-density lipoprotein (HDL), low-density lipoprotein (LDL), and homocysteine (Hcy) were measured in the Department of the Clinical Laboratory in our hospital. Smoking and vascular risk factors (such as hypertension, diabetes mellitus, and dyslipidemia) were defined as described in previous studies (Yu et al., 2017, 2019). We also evaluated the National Institutes of Health Stroke Scale (NIHSS) at admission and the modified Rankin Scale (mRS) score at 90 days after stroke onset of all patients.

### Sample Preparation

First, 100 μl of plasma sample was placed in a 1.5-ml centrifuge tube, deproteinized with 400 μl of acetonitrile and methanol mixture (1:1, v/v), vortexed for 30 s, sonicated for 10 min, and centrifuged at 12,000 rpm for 15 min at 4°C, and the supernatants of which were collected and transferred to another 1.5-ml centrifuge tube and dried with nitrogen. Then, the sample was dissolved with 100 μl of acetonitrile and water (1:1, v/v), vortexed for 30 s, sonicated for 5 min, and centrifuged at 12,000 rpm for 15 min at 4°C. Finally, the supernatant was transferred into autosampler vials.

### Untargeted Metabolic Profiling

The AB SCIEX TripleTOF 5600 system (AB SCIEX, Foster City, CA, USA) was used for the LC-MS/MS analysis. The chromatographic separations were carried out using an ACQUITY UHPLC T3 column (100 × 2.1 mm; 1.8 μm). Mobile phase A consisted of 0.1% of aqueous formic acid and mobile phase B consisted of 0.1% of formic acid in acetonitrile. The injection volume was 2 μl for positive and negative ESI modes. The gradient elution program was set as follows: 5% B (0–2 min), 5–70% B (2–5 min), 70–90% B (5–14 min), 90–100% B (14–16 min), 100% B (16–22 min), 100% to 5% B (22–22.1 min), and 5% B (22.1–25 min). The flow rate for the mobile phase was 0.3 ml/min. The eluent was sprayed into the TripleTOF 5600 electrospray tandem mass spectrometer and analyzed in the information-dependent acquisition (IDA) mode. The conditions set for the mass spectrometer were as follows: ion source gas1, 55 psi; ion source gas2, 55 psi; curtain gas, 35 psi; ion spray voltage floating, 5,500/−4,500 (+/–) V; declustering potential, 80 V; collision energy, 40 ± 20 V; and temperature, 550°C. Dynamic background subtraction was used for screening the profile to fulfill the criteria of IDA and trace all probable minor metabolites.

### Untargeted Metabolomics Data Processing

ProteoWizard software (http://proteowizard.sourceforge.net) was used to convert the acquired LC-MS raw data into mzML format files, after which the XCMSplus software (https://sciex.com/products/software/xcms-plus-software) was employed for data processing, including peak alignment, peak area extraction, and peak retention time correction (Smith et al., 2006). Metabolites were identified using HR-MS/MS library (AB Sciex, Forster City, CA, USA) and MetDNA (http://metdna.zhulab.cn/) (Shen et al., 2019). MetaboAnalyst software (https://www.metaboanalyst.ca/) was used for metabolite data analysis. Candidate metabolites were selected based on variable influence in projection (VIP) values >1 from the partial least-squares discriminant analysis (PLS-DA) model, fold change (FC) cutoff at >1.2 (or FC <0.83), and statistical significance (*p* < 0.05).

### The Measurement of Sphingosine 1-Phosphate, MMP-9, and MCP-1

In the validation stage, the plasma levels of sphingosine 1-phosphate (S1P), MMP-9, and MCP-1 were determined by using the ELISA Kit (ELISA Kit for S1P, competitive method, Catalog No. CEG031Ge; ELISA Kit for MMP-9, double antibody sandwich method, Catalog No. SEA553Hu; ELISA Kit for MCP-1, double antibody sandwich method, Catalog No. SEA087Hu; USCN Business Co., Ltd., Wuhan, China USCN Business Co., Ltd., Wuhan, China) according to the instructions of the manufacturer.

### Statistical Analysis

Statistical analyses for Student's *t*-test, Mann–Whitney *U*-test, the chi-square test, and the logistic regression analysis were performed using SPSS version 22.0 software (IBM SPSS, Chicago, IL, USA). The Pearson's correlation analysis was calculated using GraphPad Prism version 8.0 software (La Jolla, CA, USA). Receiver operating characteristic (ROC) curves were calculated using MedCalc statistical software (MedCalc Inc., Mariakerke, Belgium).

## Results

### Characteristics of Participants in the Discovery Stage

In the discovery stage, 30 healthy control, 27 patients with AIS with PCC, and 39 patients with AIS with GCC were enrolled. There were 41 males and 25 females with an average age of 57.59 years in the AIS group and 21 men and 9 women with an average age of 60.53 years in the control group. There were higher frequencies of hypertension and diabetes mellitus, higher systolic blood pressure (SBP) and white blood cell (WBC), and lower levels of TC, TG, and HDL in the AIS group. In addition, the GCC groups had lower NIHSS scores and SBP at admission, and lower mRS scores at 90 days after stroke onset when compared with the PCC group. There were no significant differences between GCC and PCC groups regarding age, sex, and vascular risk factors. Meanwhile, diastolic blood pressure (DBP), WBC, TC, TG, LDL, HDL, and Hcy levels showed no significant difference between groups (Table 1).

**Table 1:** General characteristics of control and patients with AIS in the discovery stage.

	**Control (*n* = 30)**	**AIS patients (** ***n*** **=** **66)**			***p***	
		**GCC (*n* = 39)**	**PCC (*n* = 27)**	**Total (*n* = 66)**	**AIS vs. Control**	**GCC vs. PCC**
Age, years	60.53 ± 9.76	56.69 ± 11.82	58.89 ± 11.01	57.59± 11.46	0.226	0.448
Sex (male, *N*, %)	21 (70%)	24 (61.5%)	17 (63.0%)	41 (61.2%)	0.454	0.907
Hypertension (*N*, %)	12 (40%)	21 (53.8%)	20 (74.1%)	41 (62.1%)	0.043	0.096
Diabetes mellitus (*N*, %)	2 (6.7%)	9 (23.1%)	12 (44.4%)	21 (31.8%)	0.009	0.067
Hyperlipidemia (*N*, %)	17 (56.7 %)	20 (51.3%)	14 (51.9 %)	34 (51.5 %)	0.639	0.964
Smoking (*N*, %)	10 (33.3%)	16 (41.0%)	15 (55.6%)	31 (47.0%)	0.211	0.245
SBP, mmHg	129.97 ±15.09	137.44 ± 17.57	149.70 ± 22.72	142.46 ± 20.59	0.001	0.016
DBP, mmHg	79.70 ± 10.74	80.92 ± 12.29	81.96 ± 13.37	81.35 ± 12.65	0.537	0.745
WBC, ×10^9^/L	6.00 ± 1.23	7.25 ± 1.74	8.04 ± 3.14	7.57 ± 2.42	<0.001	0.237
TG, mmol/L	2.27 ± 1.58	1.63 ± 0.67	1.68 ± 0.81	1.64 ±0.72	0.045	0.553
TC, mmol/L	4.79± 1.26	4.33 ± 1.24	4.06 ± 1.12	4.22 ± 1.19	0.035	0.368
HDL, mmol/L	1.20 ± 0.27	1.06 ± 0.24	0.98 ± 0.22	1.02 ± 0.23	0.001	0.150
LDL, mmol/L	2.99 ± 0.77	2.72 ± 0.921	2.57 ± 0.88	2.60 ± 0.90	0.089	0.509
Hcy, μmol/L	14.72 ± 9.44	13.70 ± 5.99	15.12 ± 9.51	14.28 ± 7.59	0.806	0.459
NIHSS score at admission	–	5.79 ± 3.91	9.63 ± 7.32	7.36 ± 5.83	–	0.018
mRS score on 90 days	–	1.69 ± 0.86	2.26 ± 1.13	2.76 ± 1.18	–	0.024

*AIS, acute ischemic stroke; GCC, good collateral circulation; PCC, poor collateral circulation; SBP, systolic blood pressure; DBP, diastolic blood pressure; WBC, white blood cell; TC, total cholesterol; TG, triglycerides; HDL, high-density lipoprotein; LDL, low-density lipoprotein; Hcy, homocysteine; NIHSS, National Institutes of Health Stroke Scale; mRS, modified Rankin Scale*.

### Differential Metabolites in the Discovery Stage

**Figure 2:**
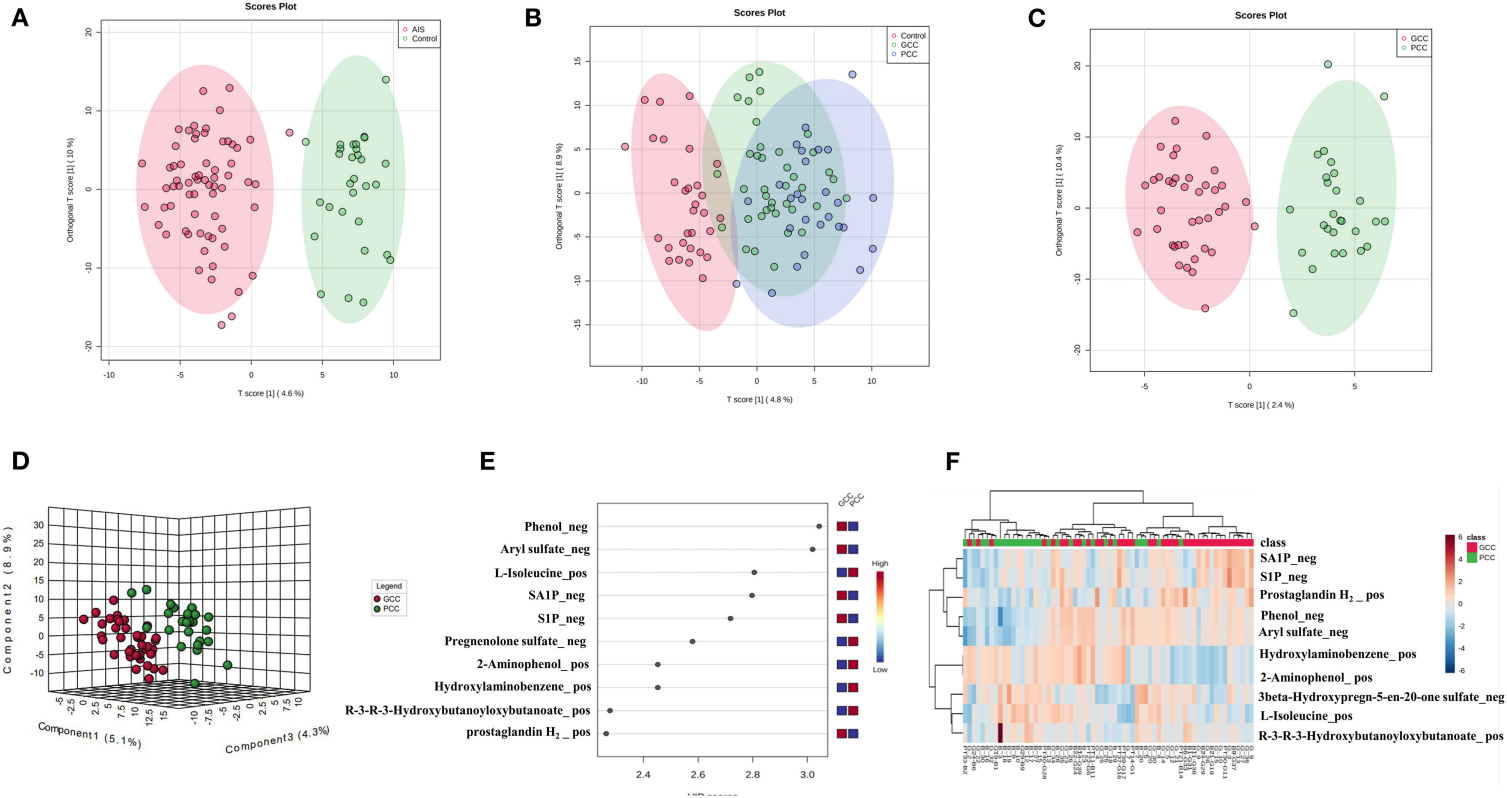
Metabolic profile of patients with GCC compared with patients with PCC. **(A)** OPLS-DA two-dimensional plot ellipses representing 95% CIs between AIS and healthy control groups. **(B)** OPLS-DA two-dimensional plot ellipses representing 95% CIs among healthy control, PCC, and GCC groups. **(C)** OPLS-DA two-dimensional plot ellipses representing 95% CIs between PCC and GCC groups. **(D)** The three-dimensional PLS-DA plot showing good model discrimination between patients with GCC compared with patients with PCC. **(E)** The VIP plot generated from the PLS-DA analysis showing the top 10 discriminative metabolites when the GCC group compared with the PCC group. **(F)** The heat map of the top 10 differentially accumulated metabolites when GCC group compared with PCC group. AIS, acute ischemic stroke; GCC, good collateral circulation; PCC, poor collateral circulation; OPLS-DA, orthogonal partial least squares discriminant analysis; PLS-DA, partial least squares discriminant analysis; VIP, variable importance in projection.

Finally, there were 571 metabolites identified in our samples. A multivariate analysis was conducted using MetaboAnalyst software. PLS-DA or the orthogonal partial least squares discriminant analysis (OPLS-DA) was used for the discriminant analysis. The OPLS-DA analysis showed good separations between control and AIS groups and GCC and PCC groups (Figures 2A,C). There was slight separation among the control, GCC, and PCC groups (Figure 2B). The PC1, PC2, and PC3 were able to account for 5.1, 8.9, and 4.3%, respectively, of the total variance by the PLS-DA analysis in the GCC group compared with the PCC group (Figure 2D).

There were 114 metabolites that distinguished patients with AIS from controls [based on FC values (FC >1.2 or FC <0.83, AIS/control), *p* < 0.05, and VIP > 1, Supplementary Table 1], and the top 10 different metabolites between control and AIS groups were ranked according to the VIP scores (Supplementary Figure 1A). The heat map of the top 10 different plasma metabolites between patients with AIS and healthy controls is shown in Supplementary Figure 1B. Thirty-seven metabolites that distinguished patients with GCC from PCC were selected using MetaboAnalyst software (Supplementary Table 4). The top 10 metabolites that distinguished the characteristic metabolic profiles of GCC and PCC were also ranked according to VIP scores as shown in Figure 2E. Differences between the samples from patients with GCC and PCC were also confirmed by comparing heat maps with the top 10 metabolites ranked (Figure 2F).

### Analyses of Metabolic Pathways

**Figure 3:**
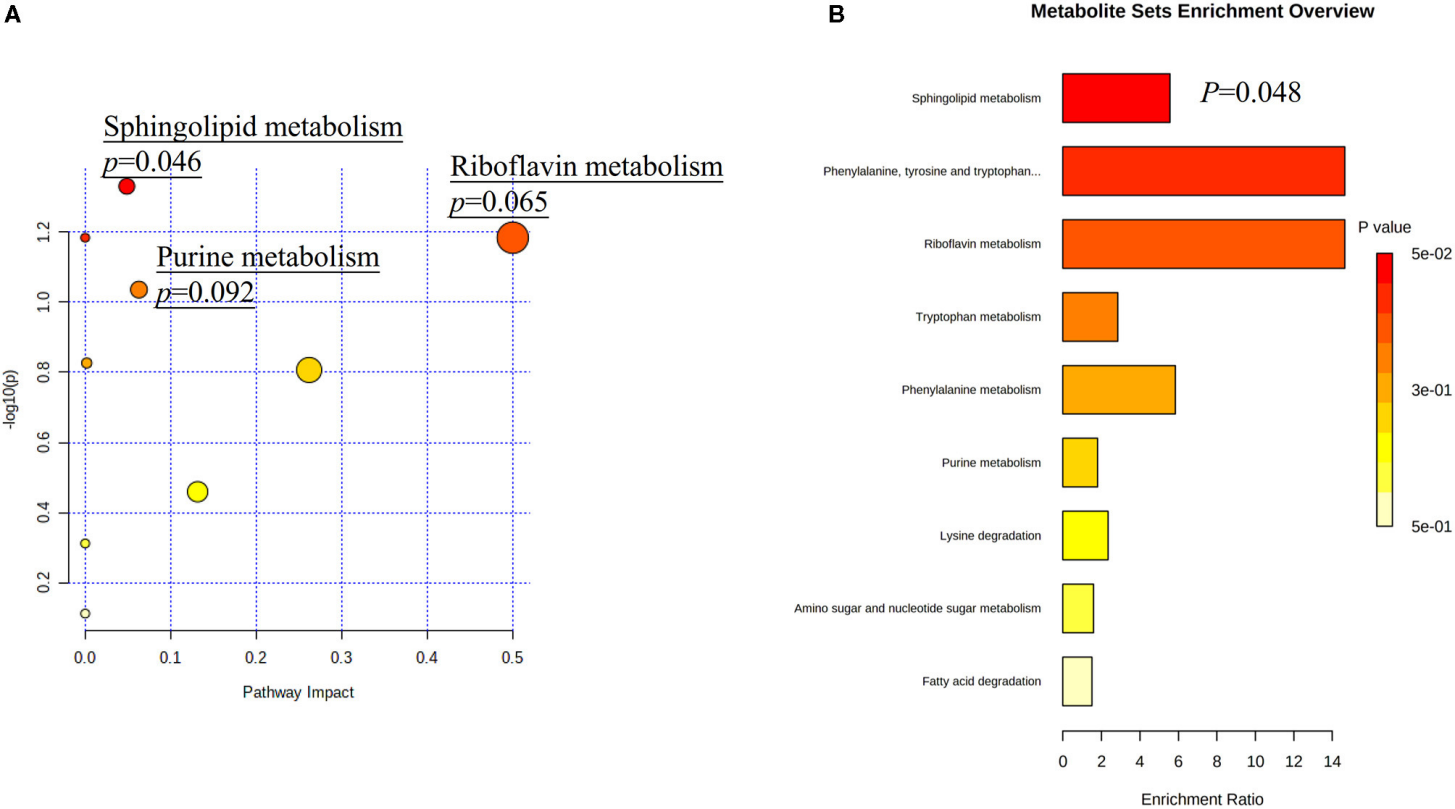
Metabolic pathway analysis based on differentially expressed metabolites identified in the plasma of patients with GCC and PCC. **(A)** Kyoto Encyclopedia of Genes and Genomes metabolic pathways database showed that the sphingolipid metabolism is the only statistically significant pathway. The *X*-axis represents the pathway impact, and the *Y*-axis represents –log10 (*p*). **(B)** Metabolite set enrichment analysis showed that the sphingolipid metabolism is the statistically significant pathway based on differentially expressed metabolites identified in the plasma of patients with GCC and PCC. GCC, good collateral circulation; PCC, poor collateral circulation.

Among all detected metabolites, 114 variables were performed searching the pathway analysis between AIS and control groups (Supplementary Table 1), and 37 variables were performed searching the pathway analysis between GCC and PCC groups (Supplementary Table 4). The identified differential metabolites were analyzed using the metabolite set enrichment analysis and Kyoto Encyclopedia of Genes and Genomes metabolic pathway database using MetaboAnalyst software. We found that arginine biosynthesis was the only statistically significant pathway on the pathway enrichment analysis between AIS and control groups (Supplementary Figures 2A,B; Supplementary Tables 2, 3), and sphingolipid metabolism was the only statistically significant pathway on the pathway enrichment analysis between GCC and PCC groups (Figures 3A,B; Supplementary Tables 5, 6).

### Identification of Potential Biomarkers

Among all detected metabolites, there were five metabolite molecules belonging to sphingolipids, including S1P_neg, S1P_pos, SA1P_neg, phytosphingosine_pos, and 3-dehydrosphinganine_pos. However, there was no significant difference in the relative plasma expression levels of these metabolites between the AIS and control groups (Supplementary Figures 3A–E).

**Figure 4:**
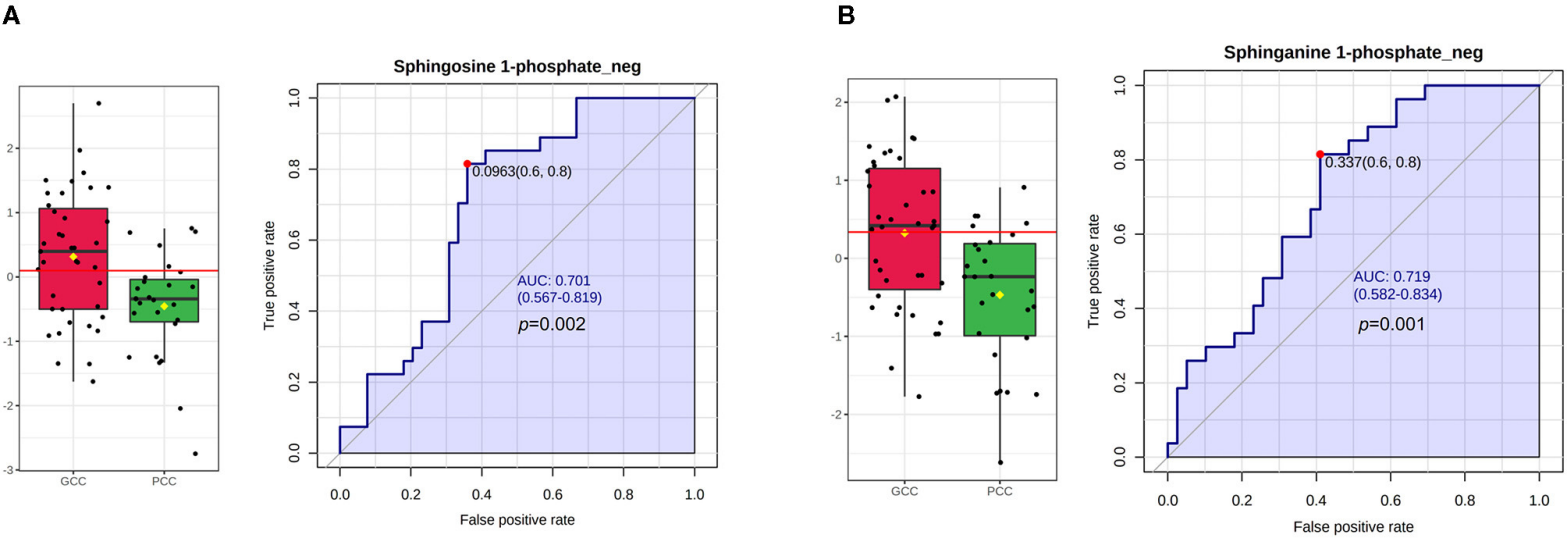
S1P and SA1P levels in GCC and PCC groups and ROC analysis in the discovery stage. **(A)** Plasma S1P_neg levels were statistically significant between GCC and PCC groups and the ROC analysis of S1P_neg in differentiating GCC and PCC groups. **(B)** Plasma SA1P_neg levels were statistically significant between GCC and PCC groups and the ROC analysis of SA1P_neg in differentiating GCC and PCC groups. GCC, good collateral circulation; PCC, poor collateral circulation; ROC, receiver operating characteristic curve; AUC, area under the curve; S1P_neg, sphingosine-1-phosphate in negative ion mode; SA1P_neg, sphinganine-1-phosphate in negative ion mode.

The relative expression levels of plasma S1P_neg and SA1P_neg were statistically higher in the GCC group than PCC group and met the condition for screening based on FC values (FC >1.2 or FC <0.83, GCC/PCC), *p* < 0.05, and VIP >1 (Figures 4A,B; Supplementary Table 4). However, the differences in plasma S1P_pos levels, phytosphingosine_pos levels, and 3-dehydrosphinganine_pos levels between patients with GCC and patients with PCC have not filled the condition for screening (Supplementary Figures 4A–C). The ROC analysis showed a good separation of GCC and PCC groups with an area under the curve (AUC) of 0.701 (95% CI: 0.567–0.819) with 81.48% of specificity and 64.10% of sensitivity for S1P_neg (Figure 4A). SA1P_neg levels demonstrated an AUC of 0.719 (95% CI: 0.582–0.834) with 81.48% of specificity and 58.97% of sensitivity (Figure 4B).

### Correlation Between the Potential Metabolites and Clinical Measures

**Figure 5:**
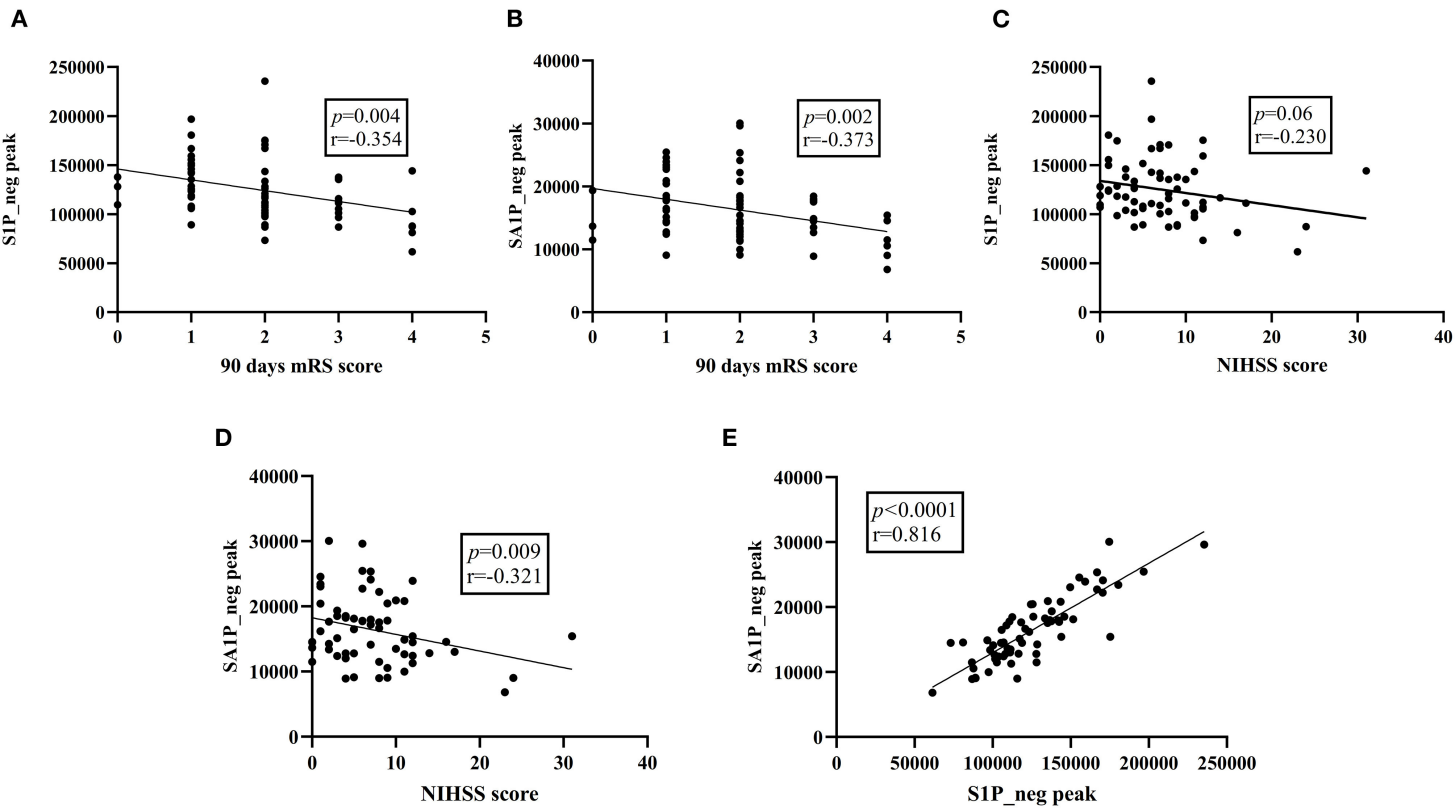
Overall correlation between differential metabolites and clinical parameters. **(A)** S1P_neg levels showed a significant negative correlation with the 90-day mRS score. **(B)** SA1P_neg levels showed a significant negative correlation with the 90-day mRS score. **(C)** S1P_neg levels showed no significant correlation with the NIHSS score. **(D)** SA1P_neg levels showed a significant negative correlation with the NHISS score. **(E)** SA1P_neg levels showed a significant positive correlation with S1P_neg levels. S1P_neg, sphingosine-1-phosphate in negative ion mode; SA1P_neg, sphinganine-1-phosphate in negative ion mode; mRS, modified Rankin Scale; NIHSS, National Institutes of Health Stroke Scale.

Moreover, in the discovery stage, we did overall correlation between differential metabolites and clinical measures using the Pearson's correlation analysis in patients with AIS (Figures 5A–E; Supplementary Figures 5A,B). S1P_neg levels showed a negative significant correlation with the 90-day mRS score (*r* = −0.354; *p* = 0.004), without correlation with NIHSS scores (*r* = −0.230; *p* = 0.06) and SBP (*r* = −0.047; *p* = 0.709). Plasma SA1P_neg levels showed a negative significant correlation with the 90-day mRS score (*r* = −0.373; *p* = 0.002) and NIHSS scores (*r* = −0.321; *p* = 0.009). There was no correlation between SA1P_neg and SBP (*r* = −0.161; *p* = 0.196). SA1P_neg levels showed a positive significant correlation with S1P_neg levels (*r* = 0.816; *p* < 0.001).

### Sphingosine-1-Phosphate Levels of the Validation Patients

In the validation stage, 27 healthy controls, 24 patients with PCC, and 29 patients with GCC were included. The baseline characteristics of these subjects are shown in Table 2. There were 37 males and 16 females with an average age of 62.49 years in the AIS group and 16 men and 11 women with an average age of 60.30 years in the control group (Table 2).

**Table 2:** General characteristics of control and patients with AIS in the validation stage.

	**Control (*n* = 27)**	**AIS patients (** ***n*** **=** **53)**			***p***	
		**GCC (*n* = 29)**	**PCC (*n* = 24)**	**Total (*n* = 53)**	**AIS vs. Control**	**GCC vs. PCC**
Age, years	60.296 ± 8.30	62.07 ± 7.52	63.00 ± 11.40	62.49 ± 9.39	0.308	0.723
Sex (male, *N*, %)	16 (59.3%)	20 (68.97%)	17 (70.83%)	37 (68.9%)	0.345	0.883
Hypertension (*N*, %)	16 (40.7%)	24 (82.8%)	19 (79.2%)	43 (81.1%)	<0.001	0.739
Diabetes mellitus (*N*, %)	3 (11.1%)	10 (34.5%)	11 (45.8%)	21 (39.6%)	0.01	0.400
Hyperlipidemia (*N*, %)	16 (59.3%)	11 (45.8%)	19 (65.5%)	30 (56.6%)	0.820	0.150
Smoking (*N*, %)	10 (37.0%)	14 (48.3%)	12 (50.0%)	26 (49.1%)	0.307	0.901
S1P (ng/mL)	197.30 ±105.21	320.75 ± 100.82	237.21 ± 72.44	282.91 ± 97.76	0.001	0.001
MMP-9 (ng/mL)	12.70 (6.63–16.19)	11.25 (6.19–27.17)	15.90 (8.65–33.70)	13.28 (7.49–27.90)	0.311	0.491
MCP-1 (ng/mL)	61.26 (49.10–68.10)	56.63 (46.47–73.44)	73.22 (53.09–126.35)	59.65 (49.00–83.24)	0.583	0.023
SBP, mmHg	132.82 ± 14.78	151.67 ± 21.76	151.67 ± 21.76	144.75 ± 20.81	0.004	0.026
DBP, mmHg	81.89 ± 11.69	81.17 ± 12.11	89.17 ± 11.65	84.79 ± 12.46	0.317	0.019
WBC, ×10^9^/L	6.95 ± 1.99	7.25 ± 2.26	8.12 ± 2.86	7.65± 2.56	0.223	0.218
TG, mmol/L	1.98 ± 1.57	1.64 ± 0.68	1.68 ± 0.76	1.65 ± 0.72	0.318	0.511
TC, mmol/L	5.06 ±1.31	4.05 ± 0.84	4.68 ± 1.05	4.35 ± 0.97	0.008	0.021
HDL, mmol/L	1.29 ±0.33	0.97 ± 0.19	1.05 ± 0.20	1.01 ± 0.20	<0.001	0.133
LDL, mmol/L	3.18 ± 0.88	2.57 ± 0.59	3.00 ± 0.83	2.77 ±0.73	0.020	0.039
Hcy, μmol/L	13.60 ± 3.08	16.64 ± 9.78	13.73 ± 4.31	15.34 ± 7.89	0.275	0.263
NIHSS score at admission	-	6.48 ± 4.89	8.21 ± 5.54	7.26 ± 5.21	-	0.234
mRS score at 90 days	-	1.83 ± 1.19	3.04 ± 1.33	2.38 ± 1.39	-	0.001

*AIS, acute ischemic stroke; GCC, good collateral circulation; PCC, poor collateral circulation; S1P, sphingosine-1-phosphate; MMP-9, matrix metalloproteinase-9; MCP-1, monocyte chemoattractant protein-1; SBP, systolic blood pressure; DBP, diastolic blood pressure; WBC, white blood cell; TC, total cholesterol; TG, triglycerides; HDL, high-density lipoprotein; LDL, low-density lipoprotein; Hcy, homocysteine; NIHSS, National Institutes of Health Stroke Scale; mRS, modified Rankin Scale*.

When the AIS group was compared with the control group, there were higher frequencies of hypertension and diabetes mellitus, higher levels of plasma S1P and SBP, and lower levels of TC, HDL, and LDL in the AIS group than in the control group (Table 2). S1P >244.49 ng/ml remained as the independent predictor of AIS compared with the control group after adjustment for hypertension, diabetes mellitus, and levels of SBP, TC, HDL, and LDL (Supplementary Table 7).

**Figure 6:**
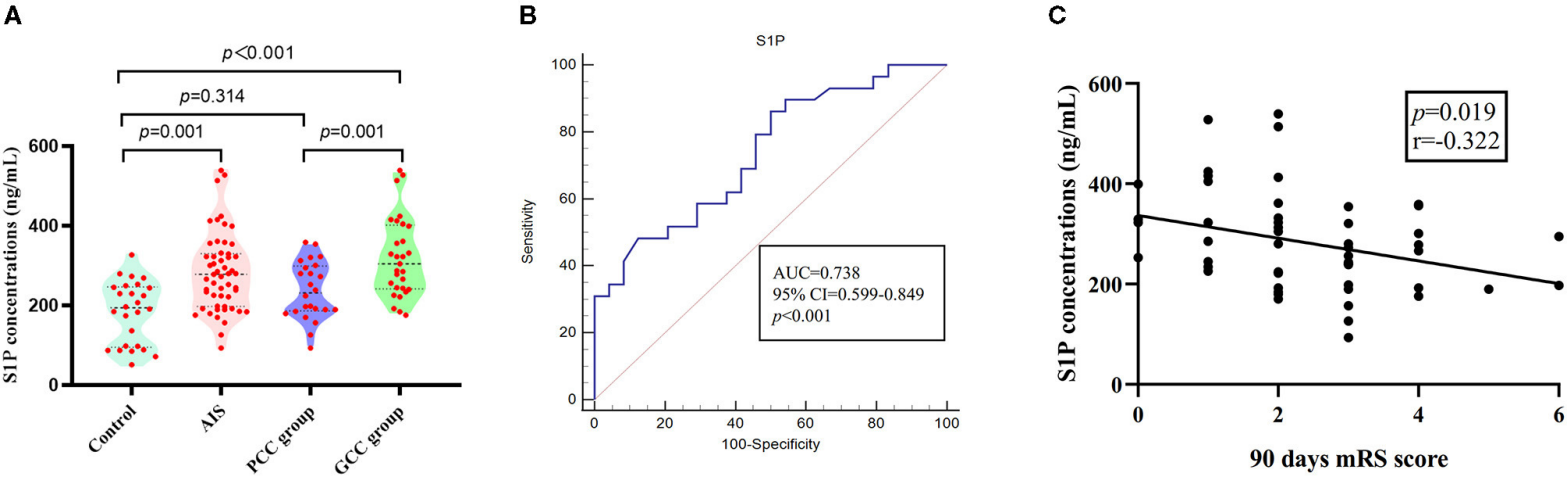
S1P levels in patients with GCC and PCC in the validation stage. **(A)** In the validation stage, there were higher plasma S1P levels in AIS groups compared with control groups; the GCC group had higher plasma S1P levels than PCC groups. **(B)** The ROC analysis of plasma S1P levels showed an AUC of 0.738 (95% CI: 0.599–0.849) to differentiate patients with GCC from PCC. **(C)** Plasma S1P levels showed a significant negative correlation with the 90-day mRS score. GCC, good collateral circulation; PCC, poor collateral circulation; ROC, receiver operating characteristic curve; AUC, area under the curve; S1P, sphingosine-1-phosphate.

When compared with patients with PCC, patients with GCC had higher levels of S1P (*p* = 0.001, Figure 6A), and plasma S1P levels demonstrated an AUC of 0.738 (95% CI: 0.599–0.849) to differentiate patients with GCC from PCC, with 86.20% of specificity and 50.00% of sensitivity (Figure 6B). High S1P levels remained as an independent predictor of GCC (compared with the PCC group) after adjusting for SBP, DBP, MCP-1, TC, and LDL levels (Table 3). In addition, we also found a negative significant correlation between the S1P levels and mRS score at 90 days in the validation cohort (Figure 6C). Plasma S1P levels showed a negative significant correlation with TG (*r* = −0.290; *p* = 0.039), without correlation with NIHSS score (*r* = −0.071; *p* = 0.612) (Supplementary Figures 6A,B).

**Table 3:** Logistic regression analysis of the association between S1P levels and cerebral collateral circulation (GCC vs. PCC).

	***P*-value**	**OR (95% CI)**
S1P (ng/mL)	0.014	1.013 (1.003–1.023)
MCP1 (ng/mL)	0.090	0.997 (0.950–1.004)
SBP, mmHg	0.962	0.999 (0.953–1.047)
DBP, mmHg	0.267	0.956 (0.883–1.035)
TC, mmol/L	0.737	0.562 (0.019–16.228)
LDL, mmol/L	0.962	1.115 (0.012–103.57)

*GCC, good collateral circulation; PCC, poor collateral circulation; S1P, sphingosine-1-phosphate; MCP-1, monocyte chemoattractant protein-1; SBP, systolic blood pressure; DBP, diastolic blood pressure; TC, total cholesterol; LDL, low-density lipoprotein*.

Since previous study found that circulating MMP-9 and MCP-1 levels were related to the opening of collateral circulation, we tested the expression of plasma MMP-9 and MCP-1 in this cohort and found no significant difference in plasma MMP-9 and MCP-1 levels between the AIS and control groups (Supplementary Figures 7, 8). When compared with patients with PCC, patients with GCC had lower MCP-1 levels (Supplementary Figure 8), but there was no statistical significance in the logistic regression analysis (Table 3).

## Discussion

In this study, we systematically analyzed the plasma metabolomic change among the AIS and control groups and patients with GCC and PCC. The metabolic profiles of plasma samples allowed the discrimination of patients with GCC and PCC. This study identified 37 metabolites that were expressed significantly differently between patients with GCC and PCC. Sphingolipid metabolism was the only significant disturbed pathway, including SA1P and S1P. In the discovery stage, compared with patients with PCC, patients with GCC had higher SA1P and S1P plasma relative levels. Besides, both SA1P and S1P had significant negative correlations with the 90-day mRS score. In the validation sample, higher plasma S1P levels were independent predictors of GCC, and plasma S1P levels demonstrated an AUC of 0.738 (95% CI: 0.599–0.849) to differentiate patients with GCC from PCC. In addition, plasma S1P levels also showed significant negative correlations with the 90-day mRS score.

Over the past decades, increasing clinical studies have convincingly established that the status of the collateral circulation affected the prognosis of ischemic stroke (Ginsberg, 2018). Good pretreatment collaterals have been reported to be correlated with favorable functional outcomes after IVT and endovascular interventions therapy (Bang et al., 2011; Leng et al., 2016). The good leptomeningeal collateral status also independently predicted favorable functional outcomes at 6 months in patients with ischemic stroke who were not receiving IVT or endovascular interventions (Lima et al., 2010). Similarly, the *post-hoc* analysis of the Warfarin versus Aspirin for Symptomatic Intracranial Disease (WASID) trial showed that the collateral status had a strong association with subsequent territorial stroke (Liebeskind et al., 2011). In this study, we used the NIHSS score to assess the severity of the stroke and found that patients with PCC had more severe neurological deficits at admission than patients with GCC. In addition, we used the mRS score to assess 90-day functional outcomes and found that patients with PCC had poorer functional outcomes. These findings were consistent with earlier studies.

**Figure 7:**
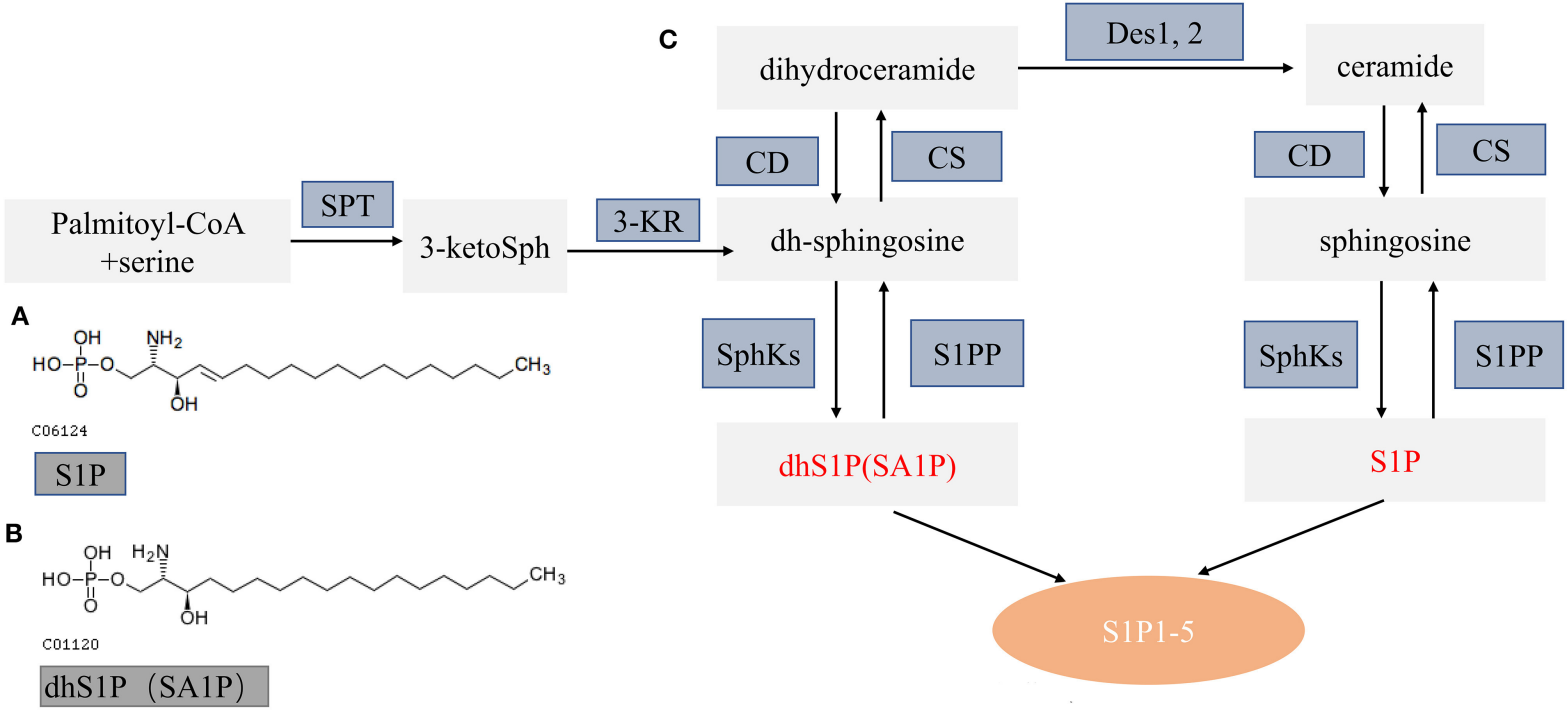
Schematic diagram of sphingolipid metabolism. **(A,B)** The molecular structure of S1P and SA1P. **(C)** Model of sphingolipid metabolism: this schematic model depicts the *de novo* pathway of sphingolipid metabolism. S1PP, sphingosine 1-phosphate phosphatase; SphKs, sphingosine kinases; S1P 1–5, S1P receptors 1–5; SPT, serine palmitoyl transferase; KR, ketosphingosine reductase; CS, ceramide synthase; CD, ceramidase; S1P, sphingosine 1-phosphate; dhS1P, dihydrosphingosine 1-phosphate; DES, dihydroceramide desaturase. Altered metabolites in this study are in red font.

It is currently recognized that sphingolipid metabolism plays a vital role in regulating various cellular processes, which are important for health and disease (Hannun and Obeid, 2018). *De novo* sphingolipid metabolism starts at the endoplasmic reticulum where condensation of serine and palmitoyl-CoA produce dihydroceramide and ceramide by several enzymatic reactions. Dihydroceramide and ceramide can be further hydrolyzed to dh-sphingosine and sphingosine and then phosphorylated to SA1P and S1P by sphingosine kinases (SphKs). Subsequently, SA1P and S1P were exported from cells to activate five specific G protein-coupled receptors (S1PR1–5) and function in diverse cell signaling pathways (Figure 7) (Callihan et al., 2012; Obinata and Hla, 2019).

To date, a mounting number of studies have demonstrated the moderating effects of the SphK-S1P-S1PR axis after stroke (Sun et al., 2016). For example, Nitzsche et al. found that endothelial S1PR1 could limit the expansion of the necrotic core in acute stroke by supporting local vasodilation (Nitzsche et al., 2021). S1P_2_ also plays an essential role in cerebral ischemia. For example, S1P_2_ could mediate M1 microglial polarization *via* the ERK1/2 and JNK pathway in the post-ischemic brain (Sapkota et al., 2019). Another study showed that the suppression of S1PR3 activity improved the neurological deficits by modulating microglial activation (Gaire et al., 2018). SphK2, mainly regulating the formation of S1P, is an important component of the sphingolipid pathway. Gene deletion studies on SphK2 showed that SphK2 was an important protective molecule after cerebral ischemia *via* attenuating ischemic brain damages (Pfeilschifter et al., 2011). FTY720, an S1PR selective agonist, can also ameliorate brain injury through multiple mechanisms after ischemic stroke (Wang et al., 2020). Zhu et al. found that those patients with AIS who received a combination of fingolimod and alteplase had lower circulating lymphocytes and smaller ischemic volumes in a multicenter randomized trial (Zhu et al., 2015). As summarized above, the SphKs/S1P/S1PRs axis is involved in the regulation of many pathophysiological processes after cerebral ischemia. In this study, we found that patients with ischemic stroke had higher plasma S1P concentration compared with controls, similar to previous findings that circulating S1P levels were higher in patients with ischemic stroke than healthy controls (Testai et al., 2014; Lucaciu et al., 2020). Besides, we found that S1P had a significant negative correlation with the 90-day mRS score, indicating that higher S1P levels might predict favorable functional outcomes. Meanwhile, we also proved that patients with GCC had higher plasma S1P levels compared with patients with PCC and healthy controls, suggesting that higher S1P levels might promote collateral circulation. Previous studies have found that circulating MMP-9 and MCP-1 levels were closely related to the opening of collateral circulation after cerebral infarction (Mechtouff et al., 2020). In this study, we found that MMP-9 and MCP-1 were not associated with the collateral circulation status after cerebral infarction, which may be attributed to the difference in the sample size and research subjects. The S1P pathway is essential for vascular development and angiogenesis (Maceyka et al., 2012). Wang et al. found that reducing the expression of SphK1 resulted in suppression of the vascular endothelial growth factor (VEGF)–SphK1–S1P signal axis-related proteins and mRNAs, further inhibiting angiogenesis in the rheumatoid arthritis model (Wang et al., 2021). Abuhusain et al. suggested that S1P concentrations were higher in glioblastomas compared with the normal brain and critical to trigger angiogenesis in glioblastoma cells (Abuhusain et al., 2013). Kiziltunç et al. found that high serum S1P levels were predictors of collateral circulation status of coronary in stable coronary artery disease (Kiziltunç et al., 2018). In the cerebral ischemia model, Nitzsche et al. found that S1P signaling played a key role in maintaining cerebral tissue perfusion and supporting vasoreactivity in the ischemic penumbra (Nitzsche et al., 2021). In a prospective and randomized clinical trial, Tian et al. found that fingolimod may preserve microvasculature function, sustain the perfusion of brain tissue, and promote retrograde reperfusion from collateral circulation (Tian et al., 2018). As summarized above, we hypothesized that the SphK1-S1P-S1PR signal axis might play a vital protective role in collateral circulation; however, the specific mechanism needs to be further explored.

SA1P, also known as dihydrosphingosine-1-phosphate (dhS1P), is produced by phosphorylation of dihydrosphingosine by sphingosine kinases (Figure 7) (Callihan et al., 2012; Magaye et al., 2019). SA1P has not been widely studied so far. Previous studies showed that SA1P treatment could protect the liver after ischemia/reperfusion *via* reducing hepatic necrosis and apoptosis and decreasing hepatic vascular permeability and expression of proinflammatory factors (Park et al., 2010a,b). Recently, studies demonstrated the reduced SA1P levels in the brains and plasma of rat models of Alzheimer's disease (Lin et al., 2017). In this study, we found that plasma SA1P levels were lower in patients with PCC compared with patients with GCC and had a negative correlation with the NIHSS score at admission and the 90-day mRS score, supporting that SA1P may be a biomarker for predicting collateral circulation, functional outcome, and disease severity.

This study had several limitations. First, since the number of cases was relatively small, the sample size needs to be expanded in future research. Second, the single-phase CTA used in this study was not the optimal approach to assess collateral circulation. Better methods of assessing collateral circulation, such as multiphase CTA or advanced perfusion software, might be more accurate. Third, due to the restricted research funding, we only validated S1P levels in another cohort, validation of other differential metabolites is needed.

## Conclusion

Metabolic profiling on plasma samples from patients with GCC and PCC has identified 37 differential metabolites. Sphingolipid metabolism was the most affected pathway associated with brain collateral circulation. Plasma S1P levels may serve as a significant predictor of the status of collateral circulation. The results could provide new insights into the treatment of cerebral collateral circulation.

## Data Availability Statement

The original contributions generated for the study are included in the article/Supplementary Material, further inquiries can be directed to the corresponding author.

## Ethics Statement

The studies involving human participants were reviewed and approved by the Ethics Committee of Xiangya Hospital of Central South University. The patients/participants provided their written informed consent to participate in this study.

## Author Contributions

JX: concept and design. FY, XF, XL, ZL, DL, YL, MW, LZ, and QH: clinical data. XF and FY: metabolomic analysis and interpretations, statistical analyses, and draft manuscript. All authors reviewed and edited the draft manuscript and approved the final manuscript.

## Conflict of Interest

The authors declare that the research was conducted in the absence of any commercial or financial relationships that could be construed as a potential conflict of interest.

## Publisher's Note

All claims expressed in this article are solely those of the authors and do not necessarily represent those of their affiliated organizations, or those of the publisher, the editors and the reviewers. Any product that may be evaluated in this article, or claim that may be made by its manufacturer, is not guaranteed or endorsed by the publisher.
