# Supplementary material for: Association of Plasma Metabolic Biomarker Sphingosine-1-Phosphate With Cerebral Collateral Circulation in Acute Ischemic Stroke
Source: Front Physiol. 2021 Aug 19;12:720672. doi: 10.3389/fphys.2021.720672 (PMC8416917; doi:10.3389/fphys.2021.720672)
Supplement: Supplementary file 1 [file Data_Sheet_1.PDF]

Figure S1. VIP plot and Heatmap of the top 10 differentially accumulated metabolites between AIS and control groups.

(A) VIP plot generated from the PLS-DA analysis showing the top 10 discriminative metabolites when AIS group compared to control group.

(B) Heatmap of the top 10 differentially accumulated metabolites when AIS group compared to control group.

Abbreviations: AIS: acute ischemic stroke; PLS-DA: partial least squares discriminant analysis; VIP: Variable importance in projection.

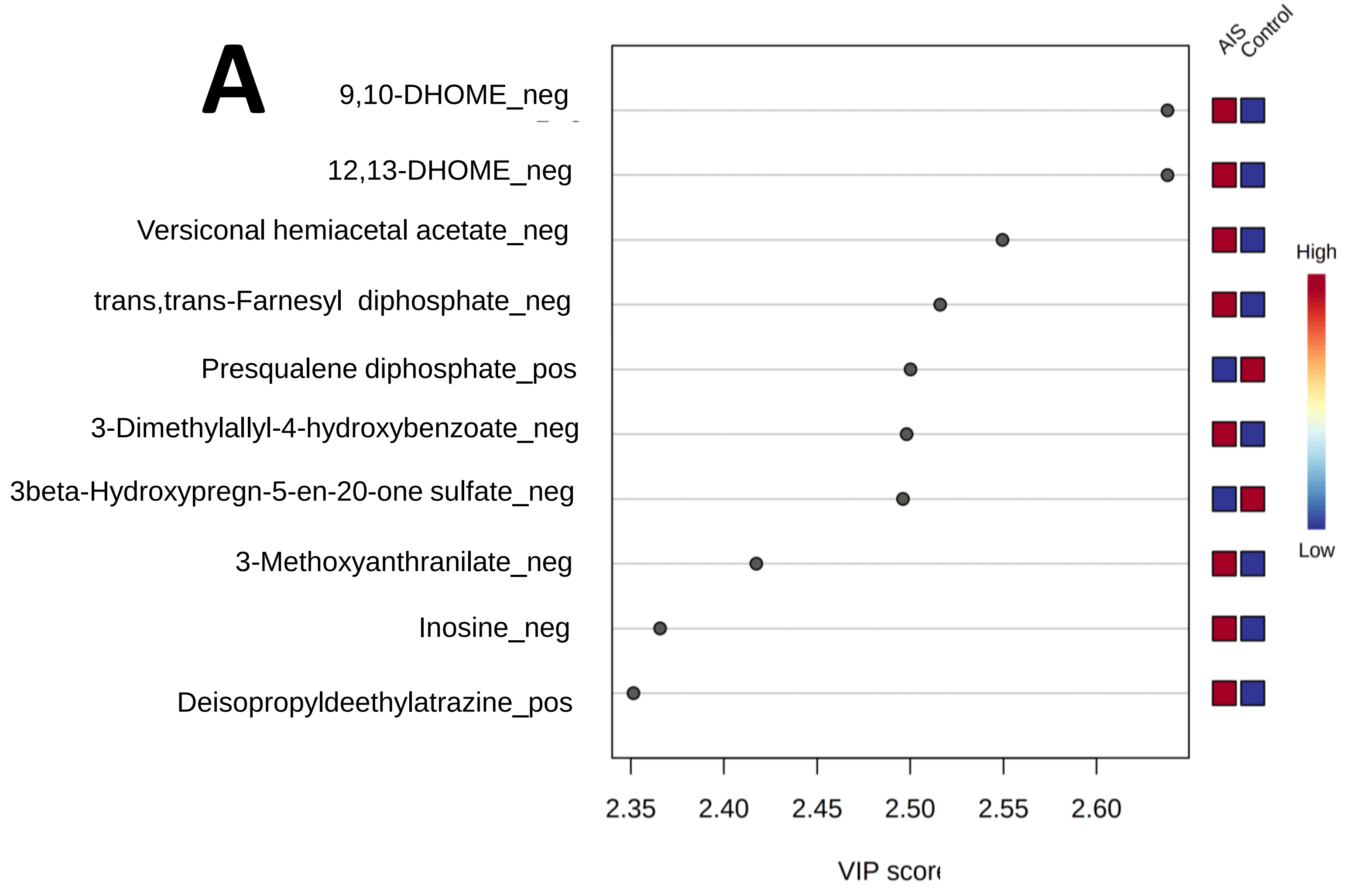

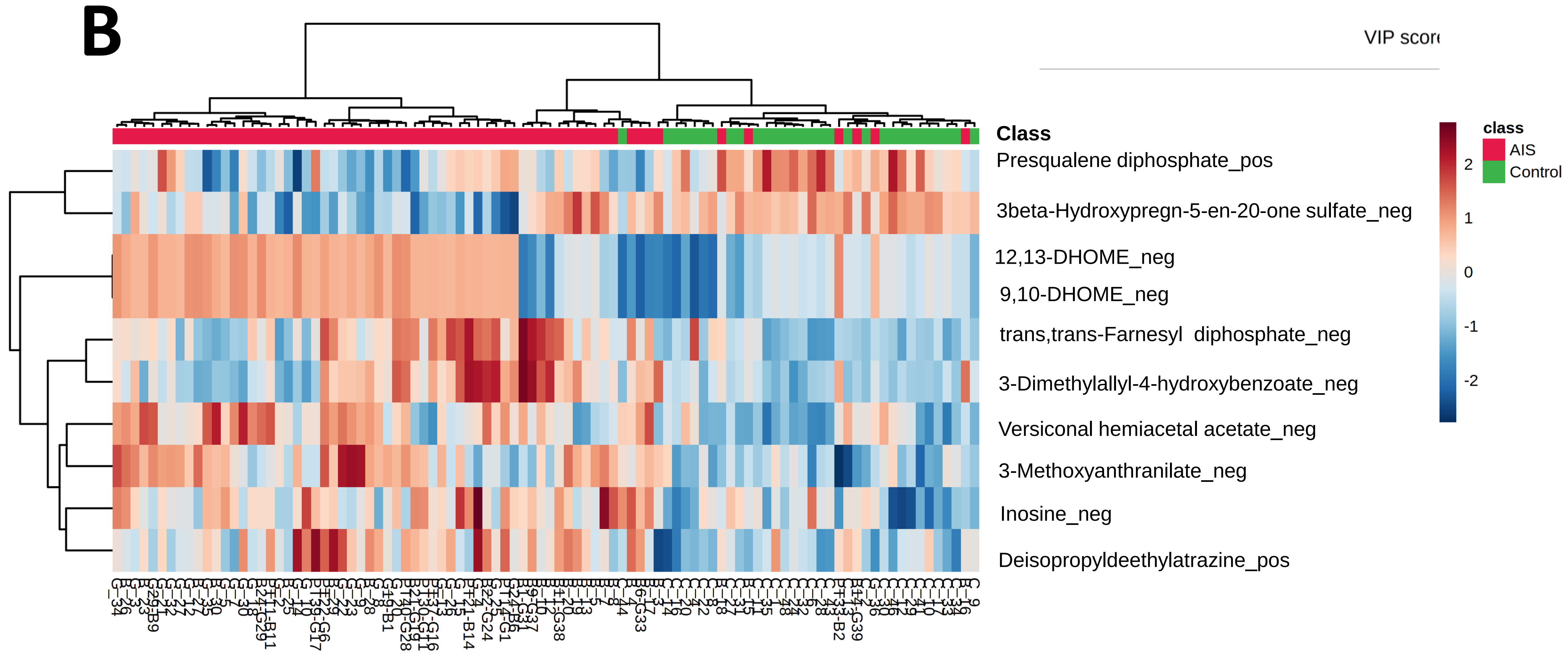

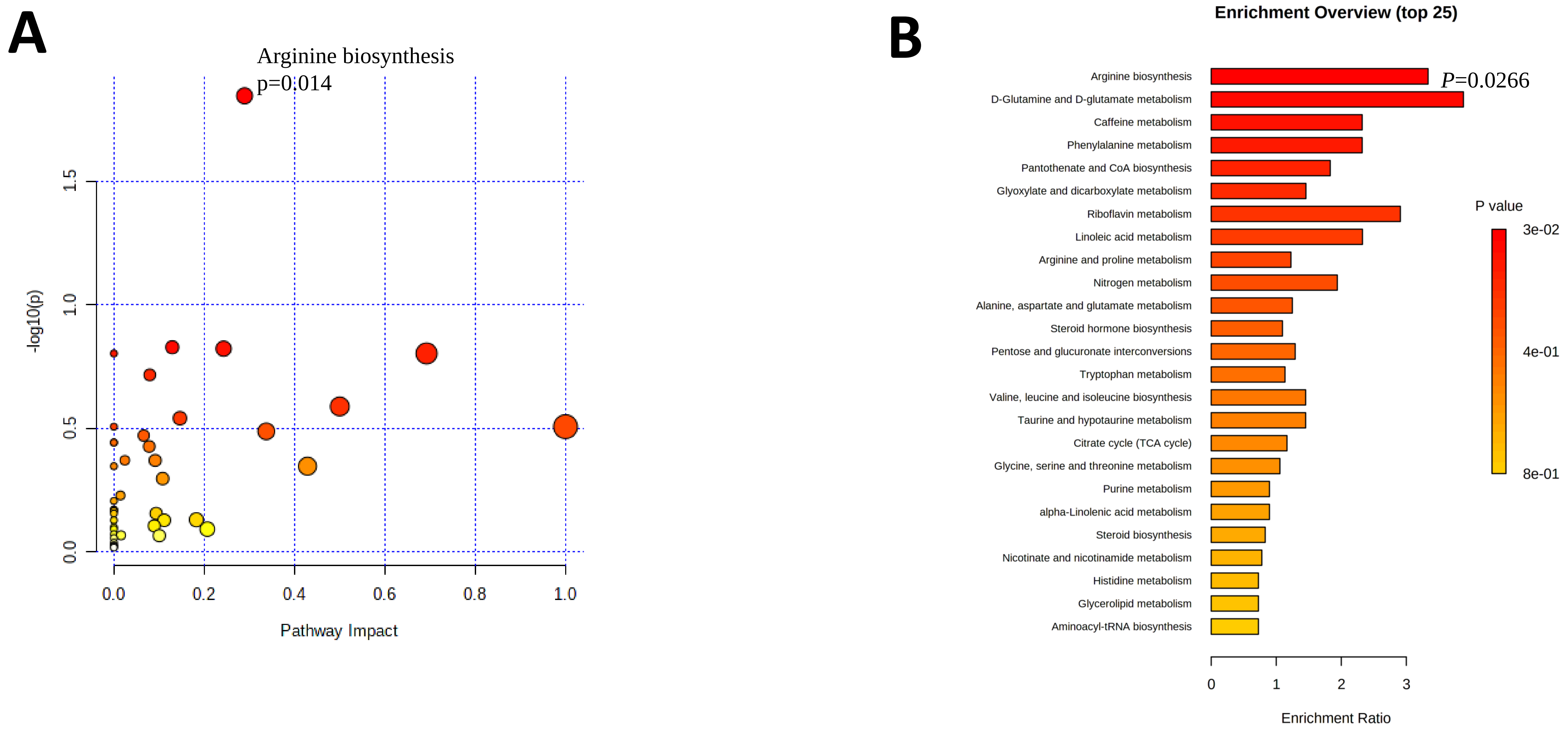

Figure S2. Metabolic pathways analysis based on differentially expressed metabolites identified in the plasma of AIS and control groups.

(A) KEGG metabolic pathways database showed that Arginine biosynthesis is the only statistically significant pathway. The X-axis represents pathway impact, and the Y-axis represents –log10 (p).

(B) Metabolite set enrichment analysis showed Arginine biosynthesis is the statistically significant pathway based on differentially expressed metabolites identified in the plasma of AIS and control groups.

Abbreviations: AIS: acute ischemic stroke.

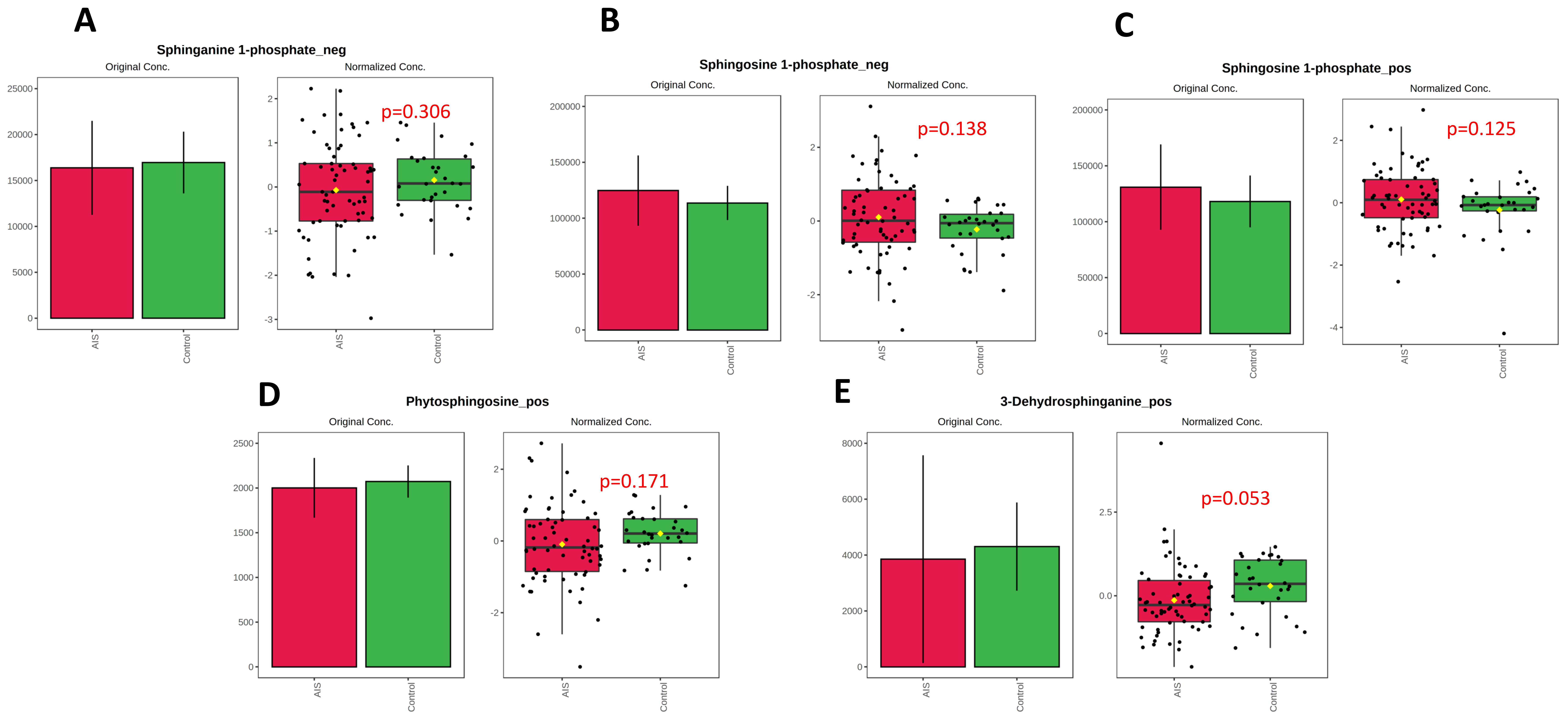

Figure S3. The relative levels of 5 sphingomyelin metabolites between AIS and control groups.
(A) Plasma SA1P_neg relative levels without statistical difference between AIS and control groups (p=0.306).
(B) Plasma S1P_neg relative levels without statistical difference between AIS and control groups (p=0.138).
(C) Plasma S1P_pos relative levels without statistical difference between AIS and control groups (p=0.125).
(D) Plasma 3-Dehydrosphinganine_pos relative levels without statistical difference between AIS and control groups (p=0.053).
(E) Plasma phytosphingosine_pos relative levels without statistical difference between AIS and control groups (p=0.171).
Abbreviations: AIS: acute ischemic stroke; S1P_neg: sphingosine-1-phosphate in negative ion mode; SA1P_neg: sphinganine-1-phosphate in negative ion mode.

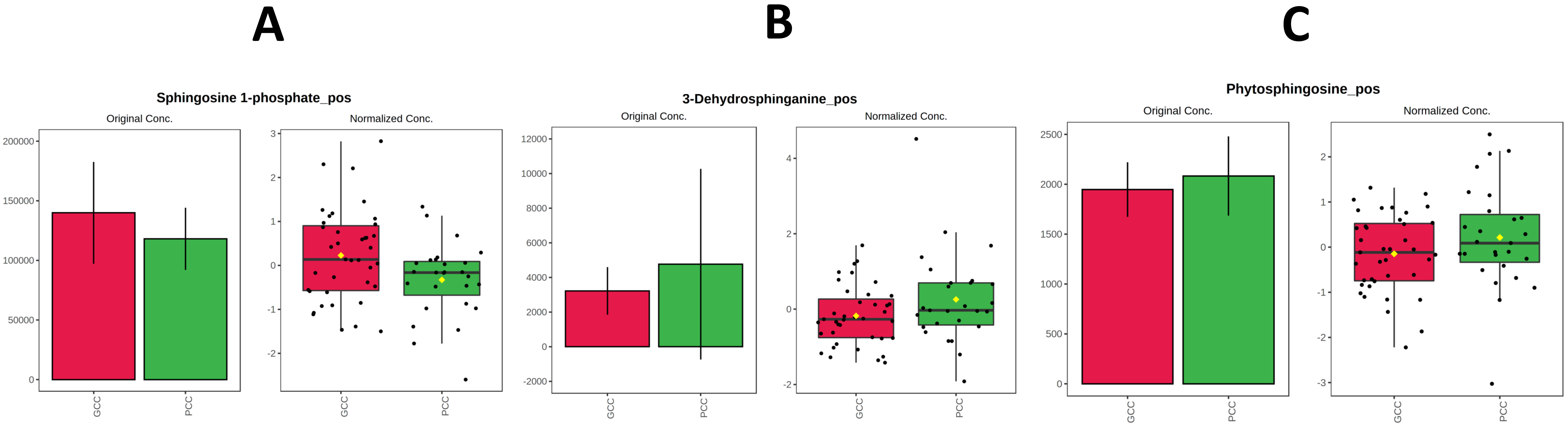

p=0.025; VIP=1.964; FC=1.184

p=0.082; VIP=1.538; FC= 0.677

p=0.150; VIP=1.277; FC=0.935

Figure S4. The relative levels of S1P_pos, phytosphingosine_pos, and 3-Dehydrosphinganine_pos in GCC and PCC groups.
(A) Plasma S1P_pos relative levels was higher in GCC group than PCC group (p=0.025; VIP=1.964; FC=1.184);
(B) Plasma 3-Dehydrosphinganine_pos relative levels without statistical difference between GCC and PCC groups (p=0.082; VIP=1.538; FC= 0.677).
(C) Plasma phytosphingosine_pos relative levels without statistical difference between GCC and PCC groups (p=0.150; VIP=1.277; FC=0.935).
Abbreviations: GCC: good collateral circulation; PCC: poor collateral circulation; VIP: Variable importance in projection; FC: fold change .

A

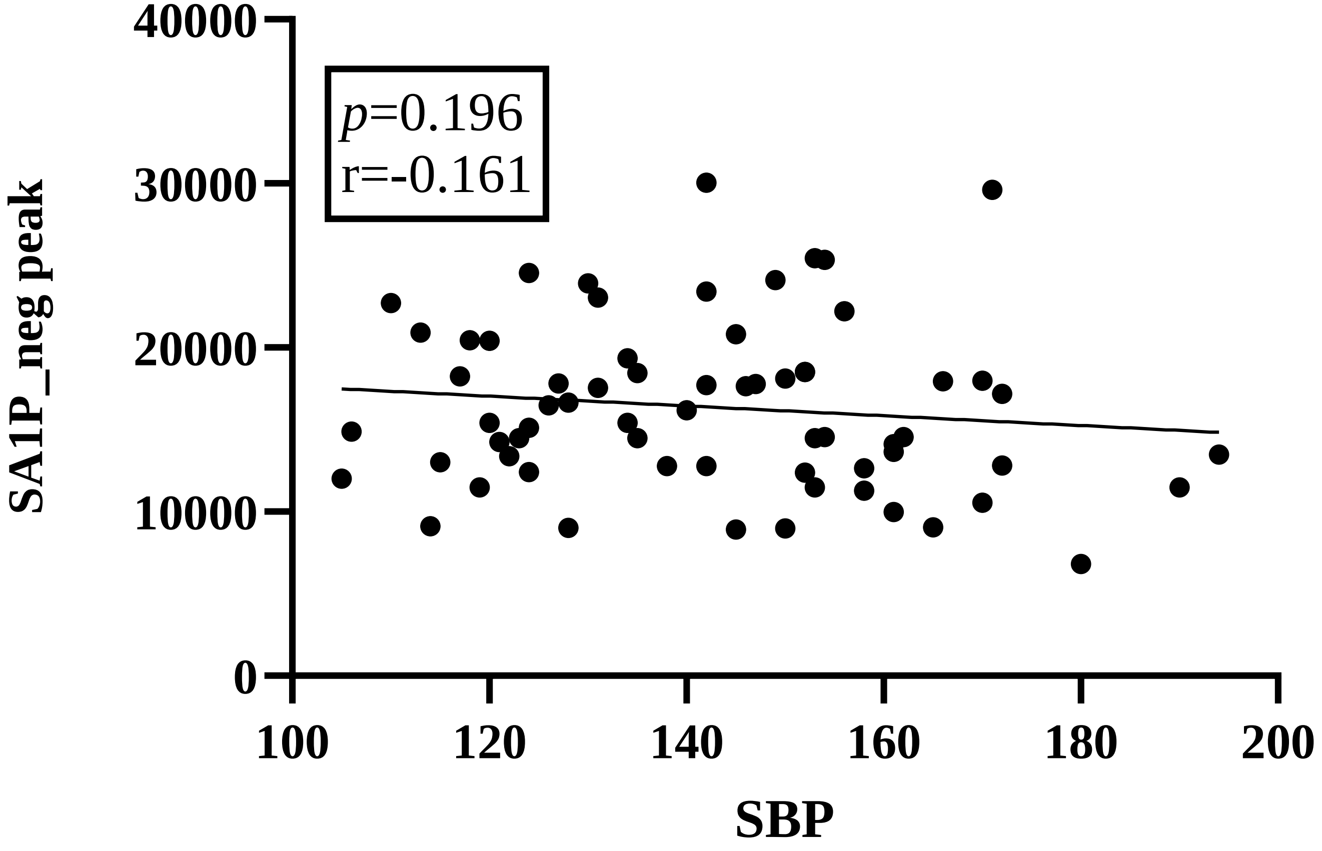

B

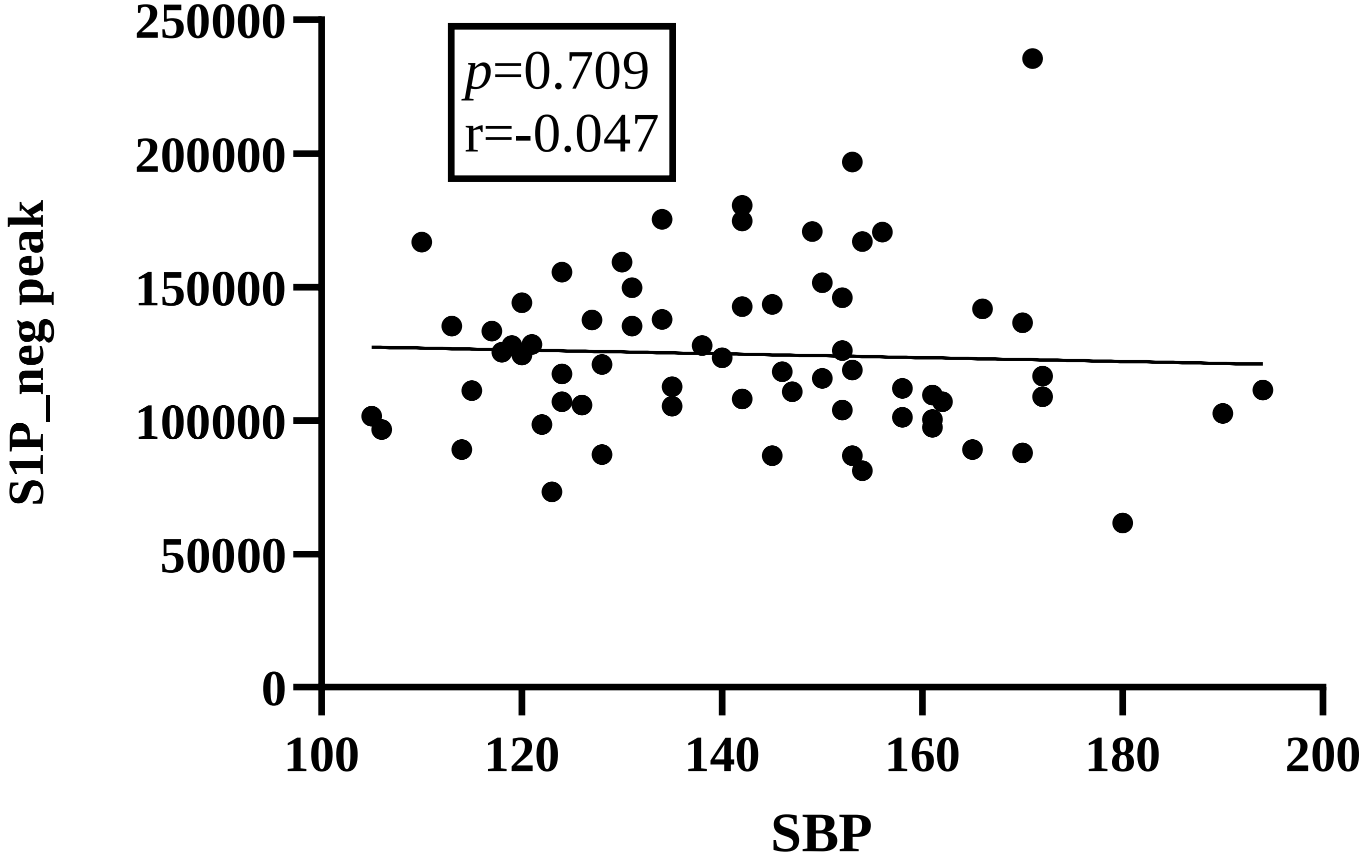

Figure S5. In discovery stage, overall correlation between differential metabolites and SBP.
(A) SA1P_neg levels showed without correlation with SBP (r =-0.161; p =0.196);
(B) S1P_neg levels showed without correlation with SBP (r =-0.047; p =0.709).
Abbreviations: S1P_neg: sphingosine-1-phosphate in negative ion mode; SA1P_neg: sphinganine-1-phosphate in negative ion mode; SBP: systolic blood pressure.

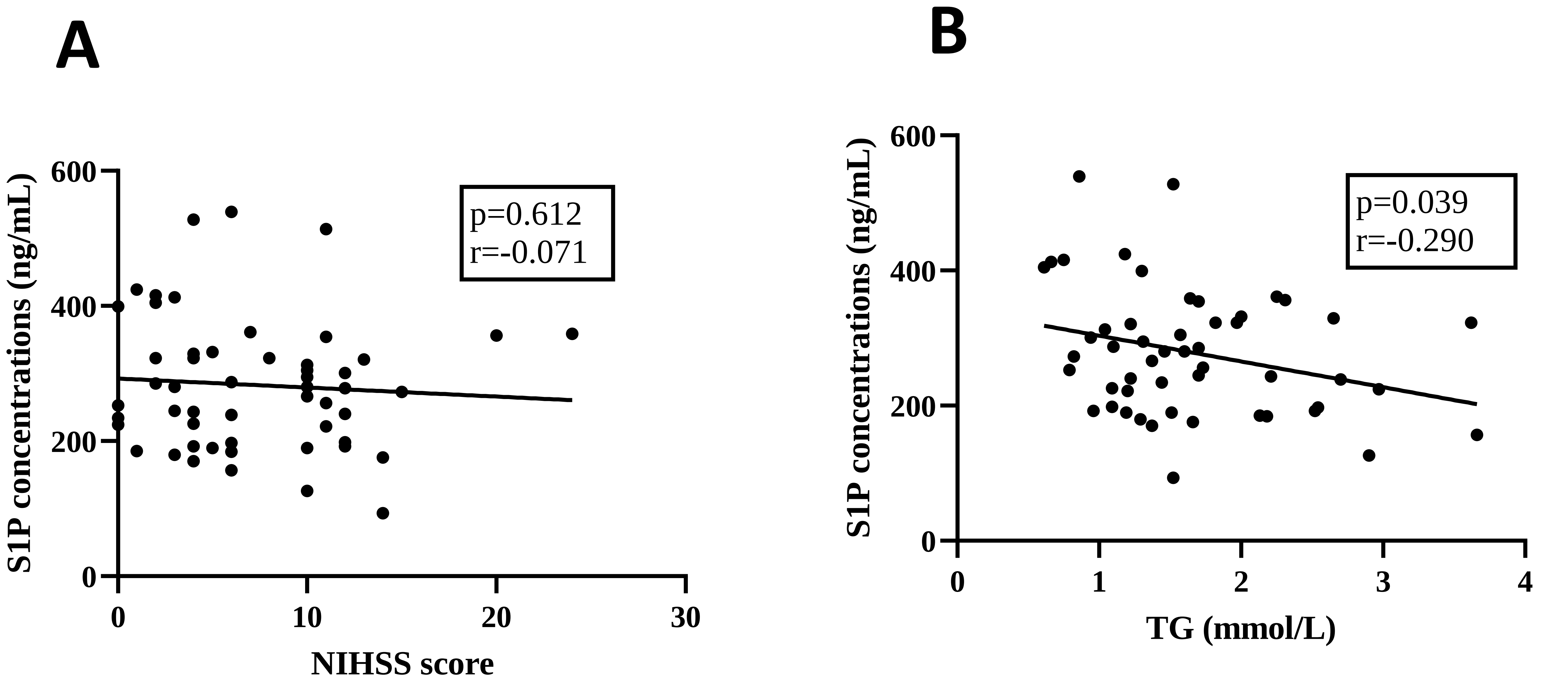

Figure S6. In validation stage, correlation analysis between Plasma S1P level and NIHSS score and TC.
(A) plasma S1P levels showed without correlation with NIHSS score (r =-0.071; p =0.612);
(B) A negative significant correlation with TG (r =-0.290; p =0.039).
Abbreviations: S1P: sphingosine-1-phosphate; TG: triglyceride; NIHSS: National Institutes of Health Stroke Scale.

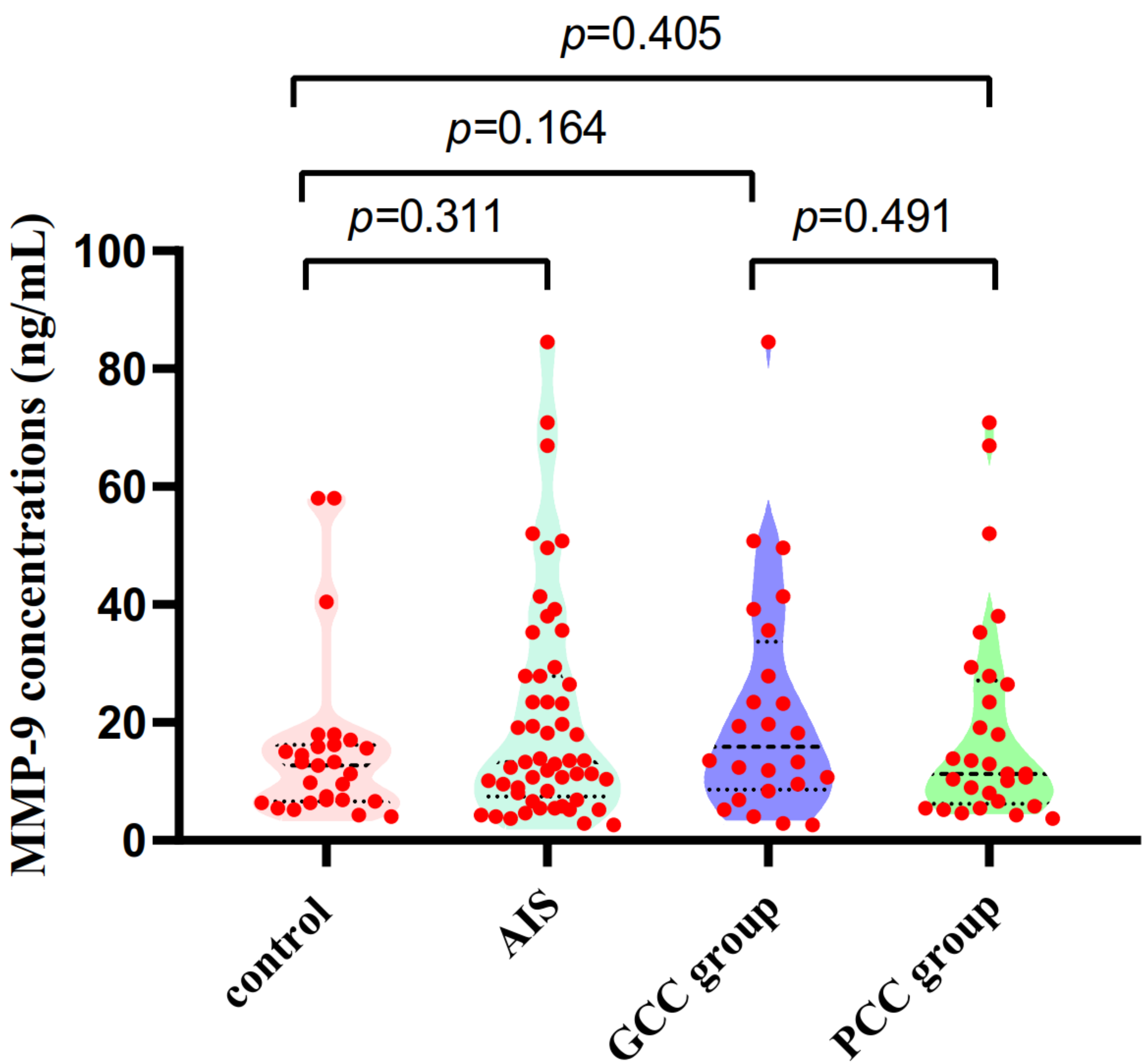

Figure S7. Plasma MMP9 levels in GCC and PCC patients in the validation stage

There was no significant difference in plasma MMP9 levels between the AIS vs control groups, and GCC vs PCC groups.

Abbreviations: AIS: acute ischemic stroke; GCC: good collateral circulation; PCC: poor collateral circulation; MMP-9: matrix metalloproteinase-9.

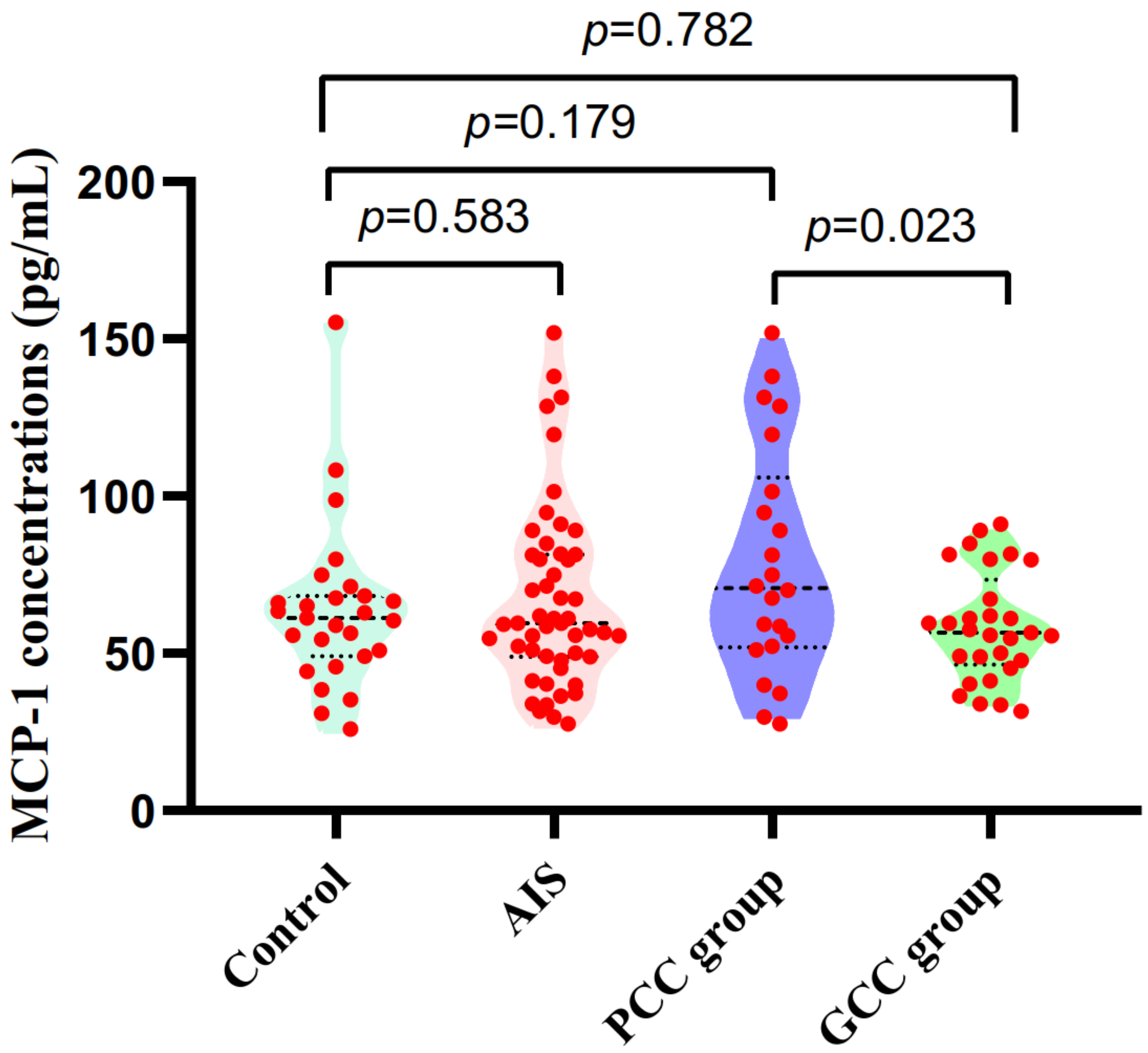

Figure S8. Plasma MCP1 levels in GCC and PCC patients in the validation stage.
There was no significant difference in plasma MCP1 levels between the AIS and control groups, when compared with PCC patients, the GCC patients had lower MCP1 levels.
Abbreviations: AIS: acute ischemic stroke; GCC: good collateral circulation; PCC: poor collateral circulation; MCP-1: monocyte chemoattractant protein-1.
