# Supplementary material for: Association of Plasma Metabolic Biomarker Sphingosine-1-Phosphate With Cerebral Collateral Circulation in Acute Ischemic Stroke
Source: Front Physiol. 2021 Aug 19;12:720672. doi: 10.3389/fphys.2021.720672 (PMC8416917; doi:10.3389/fphys.2021.720672)
Supplement: Supplementary file 2 [file Data_Sheet_2.pdf]

Table S1 Detailed list of altered metabolites between AIS patients and control.

Compound name	mz	rt	Formula	p	VIP	Fold Change	KEGG ID	Ion mode
9,10-DHOME	313.2386	761.9115	C18H34O4	6.43E-08	2.638	2.7651	C14828	Negative
12,13-DHOME	313.2386	761.9115	C18H34O4	6.43E-08	2.638	2.7651	C14829	Negative
trans,trans-Farnesyl diphosphate	381.119	451.954	C15H28O7P2	3.11E-07	2.5161	6.7933	C00448	Negative
Presqualene diphosphate	625.2841	864.761	C30H52O7P2	3.79E-07	2.5001	0.58835	C03428	Positive
Inosine	267.0735	363.325	C10H12N4O5	1.85E-06	2.3658	1.7823	C00294	Negative
L-Glutamine	145.0624	50.9035	C5H10N2O3	2.76E-06	2.3303	0.81105	C00064	Negative
cis-4-(1'-Hydroxynaphth-2'-yl)-2-oxobut-3-enoate	243.0625	476.396	C14H10O4	5.10E-06	2.2739	3.3935	C11426	Positive
4-(3-Hydroxy-2-naphthyl)-2-oxobut-3-enoic acid	243.0625	476.396	C14H10O4	5.10E-06	2.2739	3.3935	C16210	Positive
Phenylacetylglutamine	265.1183	412.1335	C13H16N2O4	5.40E-06	2.2685	2.2693	C04148	Positive
Hypoxanthine	137.046	72	C5H4N4O	1.44E-05	2.174	1.577	C00262	Positive
Phenylacetylglutamine	263.1041	409.0855	C13H16N2O4	2.47E-05	2.119	2.1268	C04148	Negative
Ornithine	131.083	53.527	C5H12N2O2	4.47E-05	2.0569	0.7411	C00077	Negative
L-Carnitine	162.112	54	C7H15NO3	5.04E-05	2.0441	1.242	C00318	Positive
p-Aminobenzoic acid	138.055	60	C7H7NO2	6.13E-05	2.0229	0.52269	C00568	Positive
Trigonelline	138.055	60	C7H7NO2	6.13E-05	2.0229	0.52269	C01004	Positive
N-Acetylputrescine	131.1175	413.0885	C6H14N2O	7.24E-05	2.0045	1.5989	C02714	Positive
3-Hydroxybutyric acid	103.04	96	C4H8O3	7.42E-05	2.0018	2.7373	C01089	Negative
L-Citrulline	174.0885	53.604	C6H13N3O3	0.000122	1.9456	0.74043	C00327	Negative
2-Amino-3-carboxymuconate semialdehyde	203.0696	476.406	C10H12O3	0.000148	1.9233	2.2996	C04409	Positive
Catechol	109.0303	387.223	C6H6O2	0.00022	1.8768	0.56194	C00090	Negative
Hydroquinone	109.0303	387.223	C6H6O2	0.00022	1.8768	0.56194	C00530	Negative
Pantetheine	277.1229	388.3895	C11H22N2O4S	0.000237	1.8678	1.2143	C00831	Negative

Aflatoxin B2	315.0795	375.577	C17H14O6	0.00026	1.8566	2.2467	C16753	Positive
3,4-Dihydroxybenzoate	153.0196	438.028	C7H6O4	0.000289	1.8436	0.62463	C00230	Negative
L-Leucine	132.1019	184.215	C6H13NO2	0.000301	1.8384	1.3789	C00123	Positive
Taurine	124.007	48	C2H7NO3S	0.000329	1.8275	0.77376	C00245	Negative
2-Hydroxy-3-phenylpropenoate	199.0072	482.643	C9H8O3	0.000332	1.8265	0.40684	C02763	Negative
Indole	173.0726	480.476	C10H10N2O	0.000389	1.8069	3.7238	C00463	Negative
2-Aminophenol	132.0441	401.588	C6H7NO	0.000463	1.7849	1.5905	C01987	Positive
1,2-Bis(4-hydroxyphenyl)-2-propanol	267.109	423.385	C15H16O3	0.000692	1.7328	1.4451	C13629	Positive
L-Aspartate	132.0306	51.119	C4H7NO4	0.000748	1.7225	1.2522	C00049	Negative
Anisole	107.0511	451.379	C7H8O	0.000937	1.6925	2.5719	C01403	Negative
Benzyl alcohol	107.0511	451.379	C7H8O	0.000937	1.6925	2.5719	C03485	Negative
Dephospho-CoA	688.1699	385.0765	C21H35N7O13P2S	0.001049	1.6771	0.61076	C00882	Positive
(Indol-3-yl) acetamide	175.0862	482.73	C10H10N2O	0.001049	1.6772	3.5286	C02693	Positive
Cortisol	422.2602	463.211	C23H32O6	0.001252	1.6528	2.1261	C00735	Positive
Salicin 6-phosphate	367.0812	492.972	C13H19O10P	0.001576	1.6206	3.5879	C06188	Positive
Stearidonic acid	277.2161	998.08	C18H28O2	0.001631	1.6158	0.71957	C16300	Positive
Corticosterone	381.174	580.003	C21H30O4	0.001676	1.6119	0.7183	C02140	Negative
p-Cumate	199.059	427.71	C10H12O2	0.002138	1.5768	1.2974	C06578	Negative
Catechol	128.0704	378.5845	C6H6O2	0.002486	1.5547	1.4175	C00090	Positive
Androsta-1,4-diene-3,17-dione	285.1878	550.191	C19H24O2	0.002633	1.5462	0.62837	C20144	Positive
Ajmaline	349.1832	395.131	C20H26N2O2	0.002731	1.5407	5.6634	C06542	Positive
20-COOH-Leukotriene B4	384.2356	440.818	C20H30O6	0.002886	1.5324	2.7049	C05950	Positive
Itaconic acid	129.019	78	C5H6O4	0.002999	1.5267	0.70708	C00490	Negative
Citraconic acid	129.019	78	C5H6O4	0.002999	1.5267	0.70708	C02226	Negative
cis-1,2-Dihydro-3-ethylcatechol	158.1171	390.093	C8H12O2	0.003188	1.5174	0.73033	C06727	Positive
Prostaglandin H2	353.2298	642.832	C20H32O5	0.00329	1.5126	1.2038	C00427	Positive

2-Fluorocyclohexadiene-cis-1,2-diol-1-carboxylate	175.0385	463.866	C7H7FO4	0.003373	1.5088	2.2586	C16482	Positive
trans-o-Hydroxybenzylidenepyruvate	191.0351	462.624	C10H8O4	0.003401	1.5075	2.108	C06203	Negative
Aniline	128.0359	363.512	C6H7N	0.003557	1.5007	1.2396	C00292	Negative
11beta,21-Dihydroxy-5beta-pregnane-3,20-dione	371.2281	401.138	C21H32O4	0.003797	1.4906	20.762	C05475	Positive
3alpha,21-Dihydroxy-5beta-pregnane-11,20-dione	371.2281	401.138	C21H32O4	0.003797	1.4906	20.762	C05478	Positive
5-Hydroxyisourate	218.997	417.795	C5H4N4O4	0.003821	1.4896	0.21788	C11821	Negative
Creatine	132.077	60	C4H9N3O2	0.004512	1.4637	1.3973	C00300	Positive
Xanthine	153.0404	178.6885	C5H4N4O2	0.004953	1.449	1.2891	C00385	Positive
5'-Benzoylphosphoadenosine	490.0495	483.936	C17H18N5O8P	0.005478	1.4328	1.5882	C06433	Positive
Glycochenodeoxycholate 7-sulfate	528.2641	588.299	C26H43NO8S	0.005819	1.4231	2.0976	C01324	Negative
(1E,3E)-4-Hydroxybuta-1,3-diene-1,2,4-tricarboxylate	203.0224	390.5395	C7H6O7	0.006439	1.4066	1.2982	C04324	Positive
(1E)-4-Oxobut-1-ene-1,2,4-tricarboxylate	203.0224	390.5395	C7H6O7	0.006439	1.4066	1.2982	C04434	Positive
CMP-N-glycoloylneuraminate	595.1291	490.398	C15H25N5O15P2	0.006689	1.4004	1.7595	C03691	Positive
4-Methylumbelliferyl acetate	217.051	490.745	C12H10O4	0.006932	1.3945	2.7402	C03837	Negative
trans-Zeatin riboside	534.0759	487.245	C15H23N5O11P2	0.006944	1.3942	1.9095	C16429	Positive
5-Hydroxy-N-formylkynurenine	287.0463	183.53	C11H12N2O5	0.00708	1.391	1.3442	C05648	Negative
Isocitrate	191.0199	134.558	C6H8O7	0.007756	1.3758	0.74461	C00311	Negative
N-Formyl-L-glutamate	198.0433	390.7945	C6H9NO5	0.008175	1.367	1.3657	C01045	Positive
4,4'-Diapolycopene	418.3396	912.243	C30H40	0.008277	1.3649	0.66572	C19797	Positive
L-2-Aminoadipate adenylate	529.0894	485.806	C16H23N6O10P	0.008707	1.3563	1.8653	C05560	Positive
Formyl-5 hydroxykynurenamine	243.0623	213.162	C10H12N2O3	0.008836	1.3538	0.7851	C05647	Negative
(9Z)-Hexadecenoic acid	253.2175	1091.07	C16H30O2	0.009005	1.3506	1.506	C08362	Negative
5-Sulfosalicylate	216.9808	383.1945	C7H6O6S	0.009295	1.3452	1.4542	C16199	Negative
UMP	323.0228	182.443	C9H13N2O9P	0.010088	1.3311	1.7004	C00105	Negative

1-(5'-Phosphoribosyl)-5-formamido-4-imidazolecarboxamide	384.0987	489.1525	C10H15N4O9P	0.010441	1.3251	2.8715	C04734	Positive
6-Aminohexanoate	132.1017	373.1175	C6H13NO2	0.011063	1.3151	1.3484	C02378	Positive
Pyruvic acid	87.009	72	C3H4O3	0.012451	1.2942	0.80565	C00022	Negative
20-Hydroxyleukotriene E4	490.197	510.279	C23H37NO6S	0.013069	1.2856	0.6415	C03577	Negative
S-(4 Methylthiobutylthiohydroximoyl)-L-cysteine	275.0487	516.185	C8H16N2O3S2	0.014825	1.2629	0.80115	C17242	Positive
Isopentenyladenosine-5'-diphosphate	518.0831	485.021	C15H23N5O10P2	0.015448	1.2554	1.8111	C16426	Positive
O-Acetylcarnitine	204.1226	102.7065	C9H17NO4	0.016062	1.2483	1.4517	C02571	Positive
(15S)-15-Hydroxy-5,8,11-cis-13-trans-eicosatetraenoate	303.2316	535.261	C20H32O3	0.016637	1.2418	0.69335	C04742	Positive
2-Hydroxyadipate	180.0873	376.484	C6H10O5	0.017399	1.2335	1.2252	C02360	Positive
(2R,3S)-2,3-Dimethylmalate	180.0873	376.484	C6H10O5	0.017399	1.2335	1.2252	C03652	Positive
Linoleate	279.2333	1114.46	C18H32O2	0.018349	1.2236	1.3586	C01595	Negative
Xylose	151.0635	373.779	C5H10O5	0.024241	1.1706	1.2462	C00181	Positive
L-Xylulose	151.0635	373.779	C5H10O5	0.024241	1.1706	1.2462	C00312	Positive
Vanillate	167.0351	463.489	C8H8O4	0.026488	1.1532	0.76733	C06672	Negative
Caffeine	195.0871	403.007	C8H10N4O2	0.026725	1.1514	0.15361	C07481	Positive
Cholesterol sulfate	465.3053	539.0965	C27H46O4S	0.027262	1.1475	0.54066	C18043	Negative
Glycocholate	464.3019	539.1315	C26H43NO6	0.027608	1.145	0.54551	C01921	Negative
ADP-5-ethyl-4-methylthiazole-2-carboxylate	579.0359	504.57	C17H22N6O12P2S	0.02803	1.142	1.4346	C20784	Positive
Paraoxon	293.0814	566.876	C10H14NO6P	0.032394	1.1129	0.82967	C06606	Positive
21-Hydroxypregnenolone	355.2327	424.7595	C21H32O3	0.033542	1.1058	1.2198	C05485	Positive
7alpha Hydroxypregnenolone	355.2327	424.7595	C21H32O3	0.033542	1.1058	1.2198	C18038	Positive
Ursodeoxycholic acid	391.285	612	C24H40O4	0.033991	1.1031	0.52072	C07880	Negative
Hyodeoxycholic acid	391.285	612	C24H40O4	0.033991	1.1031	0.52072	C15517	Negative
Riboflavin	377.1448	402.146	C17H20N4O6	0.034421	1.1005	3.7147	C00255	Positive

2-Phenylacetamide	134.0616	413.7425	C8H9NO	0.034756	1.0985	0.69739	C02505	Negative
(E)-Phenylacetaldoxime	134.0616	413.7425	C8H9NO	0.034756	1.0985	0.69739	C19714	Negative
trans-Zeatin riboside diphosphate	352.1655	425.029	C15H21N5O5	0.035989	1.0914	0.81328	C16431	Positive
Glycocholate	466.3162	539.3845	C26H43NO6	0.036747	1.087	0.58589	C01921	Positive
Theophylline	181.0716	386.187	C7H8N4O2	0.03728	1.0841	0.20806	C07130	Positive
1,7-Dimethylxanthine	181.0716	386.187	C7H8N4O2	0.03728	1.0841	0.20806	C13747	Positive
N-Acetylanthranilate	178.0545	394.782	C9H9NO3	0.037506	1.0828	0.6168	C06332	Negative
Hippurate	178.0513	413.718	C9H9NO3	0.038614	1.0767	0.66972	C01586	Negative
Pipecolic acid	130.086	72	C6H11NO2	0.039187	1.0736	0.78559	C00408	Positive
5alpha Dihydrodeoxycorticosterone	367.1966	559.084	C21H32O3	0.039627	1.0713	1.598	C18040	Negative
L-Palmitoylcarnitine	400.3422	738.3285	C23H45NO4	0.039729	1.0708	0.75861	C02990	Positive
Indolepyruvate	204.0606	390.7955	C11H9NO3	0.040985	1.0642	1.2446	C00331	Positive
Benzene-1,2,4-triol	127.0388	506.199	C6H6O3	0.041622	1.061	0.74349	C02814	Positive
7-Dehydrodesmosterol	383.3307	590.169	C27H42O	0.043958	1.0493	1.3823	C05107	Positive
Progesterone	315.236	492.35	C17H28O4	0.044559	1.0464	0.74867	C00410	Positive
(4Z,7Z,10Z,13Z,16Z,19Z)-Docosahexaenoic acid	329.2476	1092.285	C22H32O2	0.044705	1.0457	0.6268	C06429	Positive
Glyceric acid	105.019	60	C3H6O4	0.045658	1.0412	0.74266	C00258	Negative
Salicyluric acid	194.0462	440.877	C9H9NO4	0.046218	1.0386	1.995	C07588	Negative

Table S2 Result of metabolic enrichment analysis between AIS patients and control.

Pathways	total	expected	hits	Raw p
Arginine biosynthesis	14	1.2	4	0.0266
D-Glutamine and D-glutamate metabolism	6	0.516	2	0.0876
Caffeine metabolism	10	0.861	2	0.21
Phenylalanine metabolism	10	0.861	2	0.21
Pantothenate and CoA biosynthesis	19	1.64	3	0.22
Glyoxylate and dicarboxylate metabolism	32	2.75	4	0.294
Riboflavin metabolism	4	0.344	1	0.303
Linoleic acid metabolism	5	0.43	1	0.363
Arginine and proline metabolism	38	3.27	4	0.416
Nitrogen metabolism	6	0.516	1	0.418
Alanine, aspartate and glutamate metabolism	28	2.41	3	0.438
Steroid hormone biosynthesis	85	7.32	8	0.451
Pentose and glucuronate interconversions	18	1.55	2	0.468
Tryptophan metabolism	41	3.53	4	0.476
Valine, leucine and isoleucine biosynthesis	8	0.689	1	0.514
Taurine and hypotaurine metabolism	8	0.689	1	0.514
Citrate cycle (TCA cycle)	20	1.72	2	0.525
Glycine, serine and threonine metabolism	33	2.84	3	0.551
Purine metabolism	65	5.59	5	0.673
alpha-Linolenic acid metabolism	13	1.12	1	0.691
Steroid biosynthesis	42	3.61	3	0.717
Nicotinate and nicotinamide metabolism	15	1.29	1	0.742
Histidine metabolism	16	1.38	1	0.765

Glycerolipid metabolism	16	1.38	1	0.765
Aminoacyl-tRNA biosynthesis	48	4.13	3	0.798
Terpenoid backbone biosynthesis	18	1.55	1	0.804
Arachidonic acid metabolism	36	3.1	2	0.831
Biosynthesis of unsaturated fatty acids	36	3.1	2	0.831
beta-Alanine metabolism	21	1.81	1	0.851
Pyrimidine metabolism	39	3.36	2	0.864
Pentose phosphate pathway	22	1.89	1	0.864
Pyruvate metabolism	22	1.89	1	0.864
Lysine degradation	25	2.15	1	0.897
Glycolysis / Gluconeogenesis	26	2.24	1	0.906
Primary bile acid biosynthesis	46	3.96	2	0.918
Glutathione metabolism	28	2.41	1	0.921
Cysteine and methionine metabolism	33	2.84	1	0.95
Amino sugar and nucleotide sugar metabolism	37	3.18	1	0.966
Fatty acid degradation	39	3.36	1	0.971
Valine, leucine and isoleucine degradation	40	3.44	1	0.974
Tyrosine metabolism	42	3.61	1	0.978

Table S3 Result of KEGG metabolic pathways analysis between AIS patients and control.

Pathway	Total	Expected	Hits	Raw p
Arginine biosynthesis	14	1.0026	4	0.014232
Steroid hormone biosynthesis	85	6.0871	9	0.14819
Pantothenate and CoA biosynthesis	19	1.3606	3	0.15013
Phenylalanine metabolism	10	0.71613	2	0.15703
Caffeine metabolism	10	0.71613	2	0.15703
Glyoxylate and dicarboxylate metabolism	32	2.2916	4	0.19171
Riboflavin metabolism	4	0.28645	1	0.25735
Arginine and proline metabolism	38	2.7213	4	0.28715
Synthesis and degradation of ketone bodies	5	0.35806	1	0.31067
Linoleic acid metabolism	5	0.35806	1	0.31067
Alanine, aspartate and glutamate metabolism	28	2.0052	3	0.32447
Tryptophan metabolism	41	2.9361	4	0.33738
D-Glutamine and D-glutamate metabolism	6	0.42968	1	0.36019
Nitrogen metabolism	6	0.42968	1	0.36019
Pentose and glucuronate interconversions	18	1.289	2	0.37351
Glycine, serine and threonine metabolism	33	2.3632	3	0.42501
Citrate cycle (TCA cycle)	20	1.4323	2	0.42555
Valine, leucine and isoleucine biosynthesis	8	0.5729	1	0.44891
Taurine and hypotaurine metabolism	8	0.5729	1	0.44891
Purine metabolism	65	4.6548	5	0.50396
Steroid biosynthesis	42	3.0077	3	0.58991
alpha-Linolenic acid metabolism	13	0.93097	1	0.62087
Butanoate metabolism	15	1.0742	1	0.67367

Nicotinate and nicotinamide metabolism	15	1.0742	1	0.67367
Aminoacyl-tRNA biosynthesis	48	3.4374	3	0.68177
Histidine metabolism	16	1.1458	1	0.69727
Glycerolipid metabolism	16	1.1458	1	0.69727
Terpenoid backbone biosynthesis	18	1.289	1	0.73951
Biosynthesis of unsaturated fatty acids	36	2.5781	2	0.74329
Arachidonic acid metabolism	36	2.5781	2	0.74329
Pyrimidine metabolism	39	2.7929	2	0.7828
beta-Alanine metabolism	21	1.5039	1	0.79216
Pentose phosphate pathway	22	1.5755	1	0.80725
Pyruvate metabolism	22	1.5755	1	0.80725
Lysine degradation	25	1.7903	1	0.8463
Primary bile acid biosynthesis	46	3.2942	2	0.855
Glycolysis / Gluconeogenesis	26	1.8619	1	0.85749
Glutathione metabolism	28	2.0052	1	0.8775
Cysteine and methionine metabolism	33	2.3632	1	0.91615
Amino sugar and nucleotide sugar metabolism	37	2.6497	1	0.93815
Fatty acid degradation	39	2.7929	1	0.94689
Valine, leucine and isoleucine degradation	40	2.8645	1	0.9508
Tyrosine metabolism	42	3.0077	1	0.95777

Table S4 Detailed list of altered metabolites between GCC and PCC patients

Compound name	mz (ppm)	rt (min)	Formula	*p*	VIP	Fold Change	KEGG ID	Ion mode
Phenol	93.0359	402.2545	C6H6O	0.00035092	3.0433	1.7597	C15584	Negative
Aryl sulfate	172.9919	401.881	C6H6O4S	0.00039543	3.0191	1.717	C00850	Negative
L-Isoleucine	132.102	72	C6H13NO2	0.0010772	2.8059	0.64602	C00850	Positive
Sphinganine 1-phosphate	380.2569	719.209	C18H40NO5P	0.001119	2.7974	1.2971	C01120	Negative
Sphingosine 1-phosphate	378.2416	695.233	C18H38NO5P	0.0015811	2.7188	1.2236	C06124	Negative
3beta-Hydroxypregn-5-en-20-one sulfate	395.1897	700.8085	C21H32O5S	0.0028302	2.5798	0.45406	C18044	Negative
2-Aminophenol	132.0441	401.588	C6H7NO	0.0046788	2.4525	0.78723	C01987	Positive
Hydroxylaminobenzene	132.0442	421.931	C6H7NO	0.0046788	2.4525	0.78723	C02720	Positive
Xanthine	151.0265	178.516	C5H4N4O2	0.0096115	2.2567	1.3168	C00385	Positive
12,13-DHOME	313.2386	761.9115	C18H34O4	0.010227	2.239	1.4383	C14829	Negative
9,10-DHOME	313.2386	761.9115	C18H34O4	0.010227	2.239	1.4383	C14828	Negative
3-Oxo-5beta-cholanate	375.2892	674.5295	C24H38O3	0.010553	2.23	1.5383	C03070	Positive
S-Hydroxyphenylacetothiohydroximoyl-L-cysteine	269.0667	398.8475	C11H14N2O4S	0.013159	2.1654	1.5338	C17238	Negative
2-Hydroxy-6-oxo-6-4'-chlorophenyl-hexa-2,4-dienoate	251.0174	440.1615	C12H9ClO4	0.016195	2.103	1.9608	C06587	Negative
Riboflavin	377.1448	402.146	C17H20N4O6	0.01739	2.0811	0.1534	C00255	Positive
P1,P4-Bis5'-guanosyl tetraphosphate	869.0468	496.071	C20H28N10O21P4	0.017428	2.0805	1.4168	C01261	Positive
Feruloyl-CoA	966.1639	496.151	C31H44N7O19P3S	0.017476	2.0796	1.3742	C00406	Positive
N-Acetylneuraminic acid	308.099	60	C11H19NO9	0.01785	2.0731	0.68308	C19910	Negative

cis-1,2-Dihydroxy-1,2-dihydro-7-hydroxymethylnaphthalene	193.0853	443.676	C11H12O3	0.020432	2.0308	1.3046	C14104	Positive
Coumaryl acetate	193.0853	443.676	C11H12O3	0.020432	2.0308	1.3046	C20465	Positive
7-O-Acetylsalutaridinol	372.1866	401.267	C21H25NO5	0.024439	1.9735	0.035358	C05322	Positive
trans,trans-Farnesyl phosphate	325.1518	599.179	C15H27O4P	0.024782	1.969	1.2901	C20121	Positive
5-4-Acetoxybut-1-ynyl-2,2'-bithiophene	277.0381	443.7325	C14H12O2S2	0.026849	1.9428	1.3078	C04485	Positive
Feruloyl-diketide-CoA	986.1704	493.861	C33H46N7O20P3S	0.027075	1.94	1.3157	C17741	Positive
6-Phospho-2-dehydro-D-gluconate	296.9933	442.2525	C6H11O10P	0.027108	1.9396	1.6724	C01218	Positive
Z-3-Hexen-1-ol acetate	143.1063	462.25	C8H14O2	0.029635	1.91	0.42675	C19757	Positive
alpha-Oxo-benzeneacetic acid	149.0275	386.0625	C8H6O3	0.030348	1.902	0.76297	C02137	Negative
2-Formamido-N1-5'-phosphoribosylacetamidine	336.0527	547.08	C8H16N3O8P	0.030805	1.897	1.6103	C04640	Positive
Coniferyl alcohol	181.0856	547.01	C10H12O3	0.033363	1.87	1.6198	C00590	Positive
Phenylpyruvate	182.0887	547.024	C9H8O3	0.03566	1.8472	1.6623	C00166	Positive
Coniferyl acetate	223.096	546.991	C12H14O4	0.038302	1.8224	1.558	C20225	Positive
2R-2-Hydroxy-2-methylbutanenitrile	100.0762	377.837	C5H9NO	0.03991	1.808	1.5623	C18796	Positive
Pyrimidine 5'-nucleotide	294.0632	547.033	C9H14N2O7P	0.040522	1.8026	1.482	C03536	Positive
Glutaryl-CoA	882.1536	495.3955	C26H42N7O19P3S	0.040558	1.8023	1.2927	C00527	Positive
Dihydro-O-methylsterigmatocystin	341.1087	448.879	C19H16O6	0.040576	1.8021	1.2673	C03944	Positive
Indolepyruvate	204.0606	390.7955	C11H9NO3	0.045328	1.7627	0.79439	C00331	Positive
Anthraniloyl-CoA	887.1479	495.0895	C28H41N8O17P3S	0.049189	1.7332	1.2739	C02247	Positive

Abbreviations: GCC: good collateral circulation; PCC: poor collateral circulation; VIP: Variable importance in projection; Fold change: GCC/PCC.

Table S5 Result of metabolic enrichment analysis between GCC and PCC groups

Pathways	total	expected	hits	Raw *p*
Sphingolipid metabolism	21	0.359	2	0.0483
Phenylalanine, tyrosine and tryptophan biosynthesis	4	0.0683	1	0.0667
Riboflavin metabolism	4	0.0683	1	0.0667
Tryptophan metabolism	41	0.7	2	0.153
Phenylalanine metabolism	10	0.171	1	0.159
Purine metabolism	65	1.11	2	0.306
Lysine degradation	25	0.427	1	0.352
Amino sugar and nucleotide sugar metabolism	37	0.632	1	0.475
Fatty acid degradation	39	0.666	1	0.494

Table S6 Result of KEGG metabolic pathways analysis between GCC and PCC groups

Pathways	Total	Expected	Hits	Raw *p*	Impact
Sphingolipid metabolism	21	0.35226	2	0.046739	0.04868
Phenylalanine, tyrosine and tryptophan biosynthesis	4	0.067097	1	0.065489	0
Riboflavin metabolism	4	0.067097	1	0.065489	0.5
Purine metabolism	65	1.0903	3	0.092077	0.06287
Tryptophan metabolism	41	0.68774	2	0.14906	0.00179
Phenylalanine metabolism	10	0.16774	1	0.15605	0.2619
Lysine degradation	25	0.41935	1	0.34705	0.13146
Fatty acid degradation	39	0.65419	1	0.48729	0
Steroid hormone biosynthesis	85	1.4258	1	0.77207	0

Table S7 Logistic regression analysis of the association between S1P levels and AIS patients (control vs. AIS)

	P value	OR (95% CI)
Hypertension	0.112	3.016 (0.773-11.766)
Diabetes mellitus	0.026	7.054 (1.258-39.572)
S1P>244.49 (ng/mL)	0.002	8.363 (2.129 -32.853)
SBP>136.00 mmHg	0.639	1.375 (0.364-5.199)
TC>4.37mmol/L	0.539	0.447 (0.034-5.199)
HDL>1.09 mmol/L	0.074	0.247 (0.053-1.144)
LDL>2.82mmol/L	0.786	1.395 (0.126-15.46)

Abbreviations: AIS: acute ischemic stroke; S1P: sphingosine-1-phosphate; SBP: systolic blood pressure; TC: total cholesterol; HDL: high-density lipoprotein; LDL: low density lipoprotein.
